# Bone-derived PDGF-BB drives brain vascular calcification in male mice

**DOI:** 10.1172/JCI168447

**Published:** 2023-12-01

**Authors:** Jiekang Wang, Ching-Lien Fang, Kathleen Noller, Zhiliang Wei, Guanqiao Liu, Ke Shen, Kangping Song, Xu Cao, Mei Wan

**Affiliations:** 1 Department of Orthopaedic Surgery,; 2 Department of Biomedical Engineering, and; 3 The Russell H. Morgan Department of Radiology and Radiological Science, The Johns Hopkins University School of Medicine, Baltimore, Maryland, USA.

**Keywords:** Bone Biology, Vascular Biology, Microcirculation, Neurological disorders, Osteoclast/osteoblast biology

## Abstract

Brain vascular calcification is a prevalent age-related condition often accompanying neurodegenerative and neuroinflammatory diseases. The pathogenesis of large-vessel calcifications in peripheral tissue is well studied, but microvascular calcification in the brain remains poorly understood. Here, we report that elevated platelet-derived growth factor BB (PDGF-BB) from bone preosteoclasts contributed to cerebrovascular calcification in male mice. Aged male mice had higher serum PDGF-BB levels and a higher incidence of brain calcification compared with young mice, mainly in the thalamus. Transgenic mice with preosteoclast-specific *Pdgfb* overexpression exhibited elevated serum PDGF-BB levels and recapitulated age-associated thalamic calcification. Conversely, mice with preosteoclast-specific *Pdgfb* deletion displayed diminished age-associated thalamic calcification. In an ex vivo cerebral microvascular culture system, PDGF-BB dose-dependently promoted vascular calcification. Analysis of osteogenic gene array and single-cell RNA-Seq (scRNA-Seq) revealed that PDGF-BB upregulated multiple osteogenic differentiation genes and the phosphate transporter *Slc20a1* in cerebral microvessels. Mechanistically, PDGF-BB stimulated the phosphorylation of its receptor PDGFRβ (p-PDGFRβ) and ERK (p-ERK), leading to the activation of RUNX2. This activation, in turn, induced the transcription of osteoblast differentiation genes in PCs and upregulated *Slc20a1* in astrocytes. Thus, bone-derived PDGF-BB induced brain vascular calcification by activating the p-PDGFRβ/p-ERK/RUNX2 signaling cascade in cerebrovascular cells.

## Graphical abstract

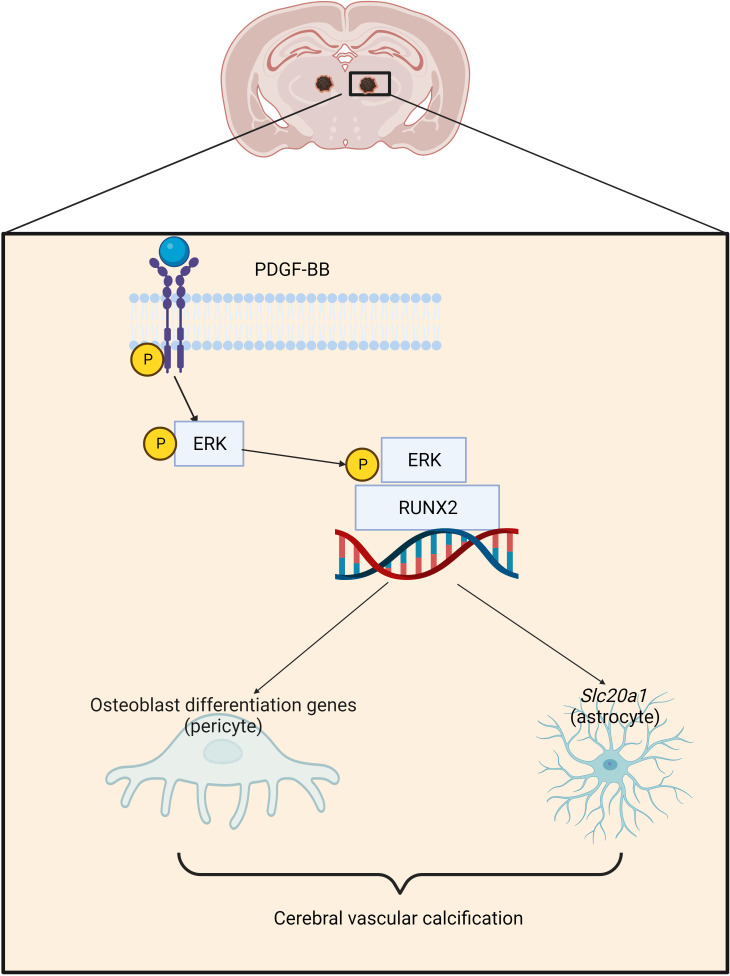

## Introduction

It has long been recognized that vascular calcification and osteoporosis often coexist in the aging population and in individuals with chronic diseases such as diabetes, dyslipidemia, hyperphosphatemia, and chronic kidney disease (1–3). The factors mediating the crosstalk between vasculature and the skeleton is a topic of intensive research. The evaluation of potential mechanisms underlying the bone-vascular crosstalk has largely focused on large vessels, such as arteries (4, 5). Arterial calcification, an independent predictor of cardiovascular disease and related mortality, is associated with decreased bone mineral density and increased risk of osteoporotic fractures (6–8). The associations have been explained by the fact that many underlying processes that regulate arterial calcification are similar to aspects of bone physiology. In bone, ecto-nucleotides pyrophosphatases/phosphodiesterases, and tissue nonspecific alkaline phosphatase (ALPL) regulate extracellular phosphate/pyrophosphate (Pi/PPi) levels to allow for regulated mineralization. Osteoblasts also secrete matrix vesicles containing enzymes, Ca^2+^, and P_i_ to facilitate mineralization of the deposited osteoid. Like the bone mineralization process, the development of arterial calcification also involves osteoblastic transdifferentiation of vascular smooth muscle cells (VSMCs) or vascular mesenchymal cells, gain-of-calcification inducers, loss-of-calcification inhibitors, and release of mineralization-competent extracellular vesicles (3, 9).

Brain calcification is much less studied relative to arterial calcification. In clinical practice, brain calcification is commonly found in elderly individuals who underwent CT imaging, which can mostly detect calcification of large arteries. The prevalence of brain calcification ranges from 1% in young individuals to over 20% in the elderly based on CT imaging (10, 11). However, the frequency of brain calcification ranges from 40% to 72% based on autopsy examinations (12), suggesting that it is an age-associated pathology. Intracranial vessel–associated calcifications have also been reported to accompany neurodegenerative diseases, such as Alzheimer’s and Parkinson’s disease, type I interferonopathies, brain tumors, and other disorders (13, 14). Brain calcification in the elderly is often detected in the pineal gland, habenula, choroid plexus, basal ganglia, falx, tentorium, petroclinoid ligaments, and sagittal sinus, whereas thalamus calcification is commonly seen under infectious conditions and metabolic disorders (15, 16). Individuals with brain calcification may or may not exhibit neurological and/or psychiatric symptoms (12, 17). The clinical manifestations include movement disorders, seizures or stroke-like events, and various cognitive and psychiatric symptoms (18–20). Of note, CT imaging can barely detect calcification of microvasculature or small-sized calcifications given the relatively low resolution of this modality. In animal studies, vascular calcification has been found in many laboratory species (21), such as mice and rats. However, age-associated brain vascular calcification and the underlying mechanisms have been largely unexplored. It appears that the thalamus is the most frequently targeted brain region for calcification in aged mice (22–24). Given that the cellular composition and characteristics of cerebral vessels are quite different from those of large arteries, investigations are needed to characterize age-associated cerebral vascular calcification and determine its pathogenic mechanisms.

The pathophysiology of primary familial brain calcification (PFBC), an autosomal inherited neuropsychiatric disorder, is better understood relative to age-associated brain vascular calcification, although a minority of brain calcifications are attributed to a genetic etiology (25, 26). PFBC is manifested by the presence of bilateral brain calcifications in the absence of secondary causes of brain calcification, and often results in psychiatric symptoms such as anxiety, cognitive impairment, and movement disorders. Mutations in 6 genes, including solute carrier 20 member 2 (*SLC20A2*), xenotropic and polytropic retrovirus receptor 1 (*XPR1*), myogenesis regulating glycosidase (*MYORG*), junctional adhesion molecule B (*JAM2*), platelet-derived growth factor B (*PDGFB*), and platelet-derived growth factor receptor β (*PDGFRB*), have been found so far to cause PFBC (21). In PFBC, mineralized deposits are located mainly in brain microvessels (e.g., brain capillaries) (27). An elegant study by Keller et al. revealed that mouse platelet–derived growth factor B (*Pdgfb*) hypomorphs (*Pdgfb^ret/ret^*) develop brain vessel–associated calcifications similar to those seen in human PFBC (28). *Pdgfb^ret/ret^* mice lack a C-terminal retention motif, which binds heparan sulfate proteoglycans. Therefore, PDGF-BB retains its receptor binding and activating ability but is diffusible.

PDGF-BB is a member of the PDGF family and is a disulfide-linked homodimeric protein composed of 2 PDGF-B chains. The PDGF family consists of the PDGF-A and -B proteins that form 3 disulfide-linked homodimers (PDGF-AA, -BB, and -AB). Both PDGF-BB and PDGF-AB can bind PDGFRβ. It is well recognized that PDGF-BB, through binding to PDGFRβ and activating downstream signaling, is essential to maintain cerebrovascular homeostasis in young healthy mice (29). The PDGF-BB/PDGFRβ signaling pathway is crucial for the maintenance of vascular homeostasis of large vessels and microvessels such as those in the cerebral vasculature (30); however, aberrantly higher circulating PDGF-BB during aging or under pathological conditions leads to vascular pathologies according to mouse studies (4, 31, 32). Our previous work revealed that TRAP^+^ preosteoclasts in bone secrete PDGF-BB, which is one of the main sources of PDGF-BB in blood circulation (33, 34). We recently found that serum PDGF-BB concentration is elevated 2- to 3-fold during aging and that skeletal preosteoclasts are a main cell type contributing to the aberrantly elevated circulating PDGF-BB (4, 32). Moreover, we identified that elevated circulating PDGF-BB is both sufficient and required to mediate age-associated large-vessel pathology (i.e., arterial stiffening) (4) and hippocampal microvessel impairment (32). In the present study, we aimed to characterize the involvement of skeleton-derived PDGF-BB in cerebral vascular calcification during natural aging.

## Results

### Aged mice develop brain calcification primarily in the thalamus region.

**Figure 1:**
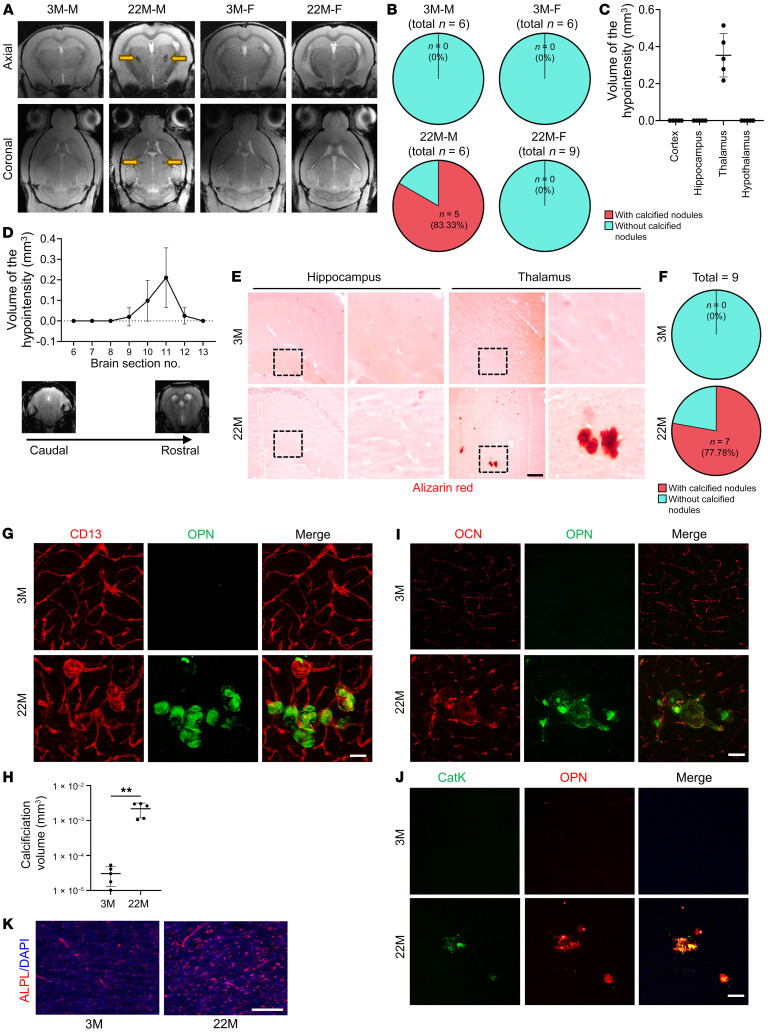
Aged male mice develop brain calcification at the thalamic regions. (**A**) Axial and coronal SWI sequence images of brains from 3-month-old male mice (3M-M), 22-month-old male mice (22M-M), 3-month-old female mice (3M-F), and 22-month-old female mice (22M-F). Calcifications (yellow arrows) were observed as black structures in the thalamic region on susceptibility-weighted images. *n* = 6–9. (**B**) Calcification incidence calculation based on the SWI analysis in **A**. (**C**) The volume of calcification in different brain regions, including the cortex, hippocampus, thalamus, and hypothalamus, in 22-month-old male mice was determined on the basis of SWI analysis. *n* = 5. (**D**) Quantification of the volume of calcification in individual serial sections (750 μm thick) by SWI throughout the entire thalamus, from caudal to rostral aspects. *n* = 5. (**E**) Alizarin red staining of brain tissue sections from 3- and 22-month-old male mice. Boxed areas are shown at higher magnification (×10) in the corresponding panels on the right. The calcification nodule is shown in red. *n* = 9. (**F**) Calculation of the calcification incidence in 3- and 22-month-old male mice based on the histology stainings. *n* = 9. (**G**) Double-immunofluorescence staining of frozen brain tissue sections from 3- and 22-month-old male mice using antibodies against CD13 and OPN. *n* = 5. (**H**) Quantification of the volume of the OPN^+^ calcified nodules in **G**. *n* = 5. The Imaris 3D reconstruction method was used to quantify the number and volume of calcification. (**I** and **J**) Double-immunofluorescence staining of frozen brain tissue sections from 3- and 22-month-old male mice using antibodies against OPN and OCN (**I**) or OPN and CatK (**J**). *n* = 3. Scale bars: 100 μm (**G**, **I**, and **J**). (**K**) Immunofluorescence staining was performed on brain sections from male mice at 3 and 22 months of age using an antibody against ALPL. *n* = 3. Scale bar: 100 μm. Data are shown as the mean ± SD. **P < 0.01, by unpaired, 2-tailed Student’s *t* test (**H**).

Brain calcification is commonly detected in elderly individuals. We evaluated whether aged mice also develop brain calcification by susceptibility-weighted imaging (SWI) (35), which is an advanced in vivo imaging technique for the detection and quantification of mouse brain calcification, given the associated high sensitivity and accuracy of this imaging modality (36, 37). Susceptibility-weighted images of male mice showed hypointensities only in the thalamic region of 22-month-old male mice (Figure 1A, yellow arrows). No hypointensity areas were found in 3-month-old male mice. Phase map analysis revealed similar findings (Supplemental Figure 1A, yellow arrows; supplemental material available online with this article; https://doi.org/10.1172/JCI168447DS1). Five of 6 (83.3%) 22-month-old male mice showed hypointensities in the thalamus (Figure 1B). Surprisingly, we detected no hypointensities in any brain regions of female mice at 3 months or 22 months of age (Figure 1, A and B). Quantification of the total calcification load in the aged male mice revealed an average of 0.3531 ± 0.161 mm^3^ volume of brain calcification (Figure 1C). The calcification loads of the 5 samples were located at the same thalamic region (i.e., slices 8–13 from the caudal side of the thalamus) (Figure 1D). To cross-validate the finding of hypointensities by SWI MRI, we performed micro-CT (μCT) scanning of the samples. Hyperdense lesions were indeed detected only in the thalamus of the aged male mice (Supplemental Figure 1B). There were no hyperdense lesions found in 3-month-old male mice or in female mice at either age. Furthermore, immunofluorescence staining of tissue sections from the samples with hypointensities identified by SWI revealed multiple nodules in thalamus that highly expressed the bone matrix protein osteopontin (OPN) (Supplemental Figure 1C), a well-established marker of brain calcification (35, 38). Thus, the hypointensity lesions detected by SWI scanning were indeed brain calcification.

We conducted a more comprehensive characterization of the brain calcification using male mice at the ages of 3 months and 22 months. Consistent with the SWI results, Alizarin red staining of brain tissue sections detected clusters of calcified nodules in the thalamus of 22-month-old mice but not in that of 3-month-old mice (Figure 1E). Calcified nodules were detected in the thalamus region in 7 of 9 mice at the age of 22 months, but none of the 3-month-old young mice developed thalamic calcification (Figure 1F). Moreover, OPN was highly expressed in these lesions in the thalamus (Figure 1G). Costaining with the blood vessel pericyte (PC) marker CD13 confirmed that the OPN-stained aggregates were associated with vessels (Figure 1G). Quantification of the calcification volume revealed dramatic differences between young and old mice (Figure 1H). In addition, co-immunofluorescence staining showed that OPN was well localized with the osteoblast-secreted protein osteocalcin (OCN) (Figure 1I) and the osteoclast-secreted protein cathepsin K (CatK) (Figure 1J) in the calcification lesions. Vessel-associated calcifications have previously been found in the thalamus in old mice with increased expression of ALPL (39), which is an enzyme that regulates mineralization in bone and other tissues. We also detected an increase in ALPL expression in the thalamus of aged mice relative to expression levels in young mice (Figure 1K). Taken together, these results demonstrate that brain vessel–associated calcifications develop in male mice during natural aging and that the lesions were located primarily in a specific thalamic region.

### Overexpression of PDGF-BB in preosteoclasts is sufficient to induce brain calcification.

**Figure 2:**
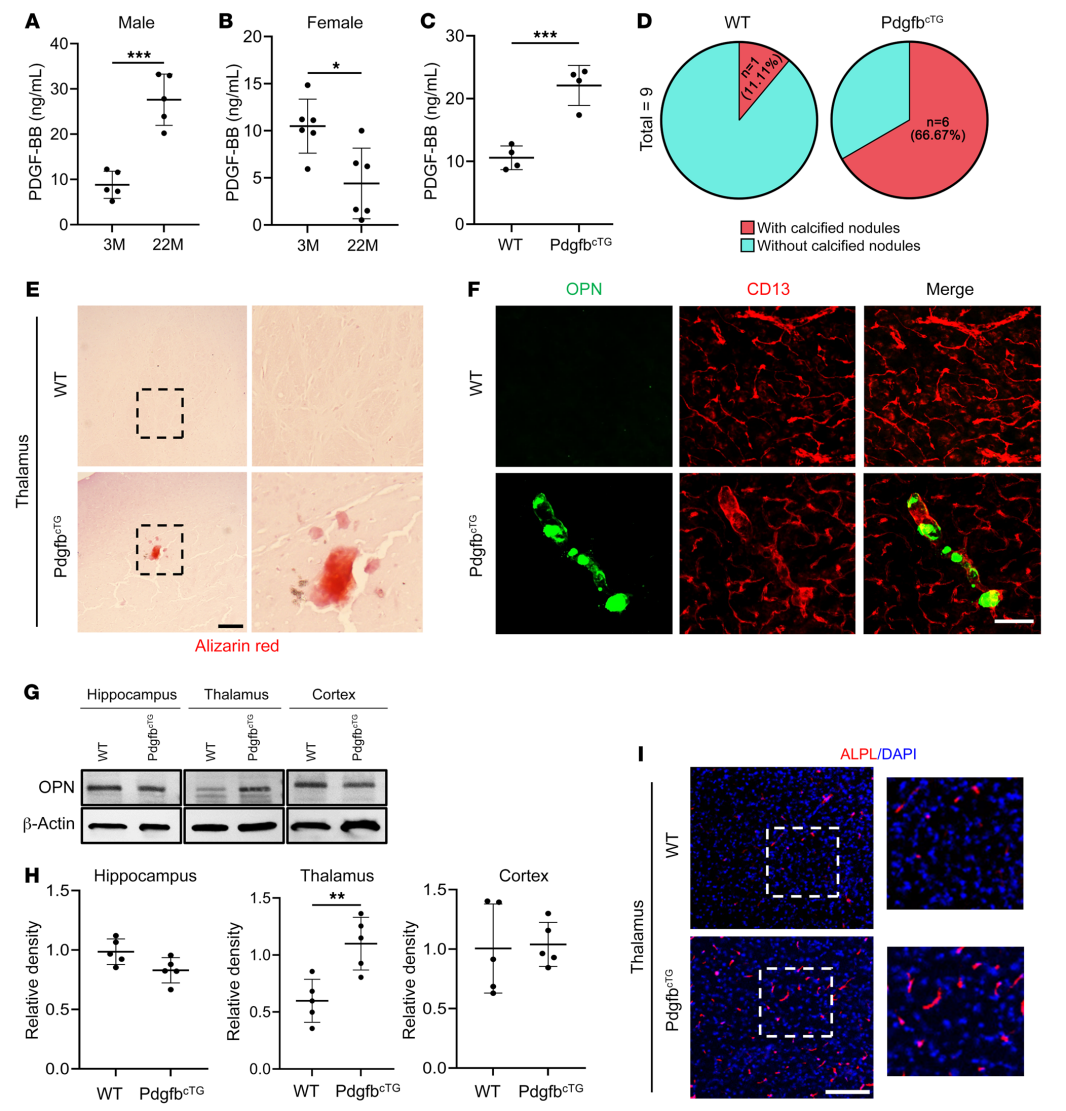
Preosteoclast-secreted PDGF-BB is sufficient to induce brain calcification. (**A**–**C**) ELISA measurements of serum PDGF-BB concentrations in 3- and 22-month-old male mice (*n* = 5) (**A**), 3- and 22-month-old female mice (*n* = 6) (**B**), and 6-month-old Pdgfb^cTG^ mice and WT littermates (*n* = 4) (**C**). (**D**) Calculation of calcification incidence in 6-month-old Pdgfb^cTG^ mice and WT littermates. *n* = 9. (**E**) Representative images of Alizarin red staining of brain tissue sections from 6-month-old Pdgfb^cTG^ mice and WT littermates. The calcification nodule is shown in red. Boxed areas are shown at a higher magnification (×10) in the corresponding panels on the right. *n* = 9. (**F**) Double-immunofluorescence staining of frozen brain tissue sections from 6-month-old Pdgfb^cTG^ using antibodies against CD13 and OPN. *n* = 3. (**G**) OPN expression in hippocampus, thalamus, and cortex from Pdgfb^cTG^ mice and WT littermates was measured by Western blot analysis. *n* = 5. (**H**) The relative intensity from **G** was calculated using ImageJ. *n* = 5. (**I**) Immunofluorescence staining of frozen brain tissue sections from Pdgfb^cTG^ mice and WT littermates using antibodies against ALPL. *n* = 3. Boxed areas are shown at higher magnification (×5) in the corresponding panels on the right. *n* = 3. Scale bars: 100 μm (**E** and **I**); 200 μm (**F**). Data are shown as the mean ± SD. *P < 0.05, **P < 0.01, and ***P < 0.001, by unpaired, 2-tailed Student’s *t* test (**A**–**C** and **H**).

We previously found that elevated circulating PDGF-BB is a key mediator for age-associated vascular pathologies (4, 32). Here, we also detected dramatically increased serum PDGF-BB concentrations in 22-month-old male mice relative to concentrations in 3-month-old male mice (Figure 2A). Relative to young (3-month-old) male mice, serum PDGF-BB levels in aged (22-month-old) male mice were nearly 3-fold higher. Surprisingly, contrary to the elevated serum PDGF-BB concentration observed in aged male mice, 22-month-old female mice exhibited lower serum PDGF-BB concentrations than did young female mice (Figure 2B). Therefore, there was a sex difference (linear regression model, sex-by-age interaction effect: *P* = 7 × 10^–7^) in the alteration of circulating PDGF-BB levels with age. To further examine whether increased serum PDGF-BB levels are involved in age-associated brain calcification, we took advantage of an established conditional *Pdgfb*-transgenic mouse line (Pdgfb^cTG^), in which *Pdgfb* is overexpressed in skeletal TRAP^+^ preosteoclasts (4, 40). PDGF-BB^+^ cells were detected in bone and bone marrow of WT littermates (Supplemental Figure 2), consistent with our previous finding that preosteoclasts are a major cell type that express PDGF-BB in bone and bone marrow (4, 33). The number of PDGF-BB^+^ cells in Pdgfb^cTG^ mice was substantially higher than in WT mice (Supplemental Figure 2), confirming the successful overexpression of PDGF-BB in the bone tissue of this conditional transgenic mouse line. Notably, serum PDGF-BB levels in Pdgfb^cTG^ mice were more than 2-fold higher relative to levels in WT mice at a young age (6 months old) (Figure 2C), mirroring the abnormality in aged male mice. In our previous study, we discovered that the elevated levels of circulating PDGF-BB in aged mice or Pdgfb^cTG^ mice were primarily generated by bone/bone marrow preosteoclasts, rather than being derived from blood myeloid cells (4). Here, we examined whether cells in brain produce excessive PDGF-BB in aged mice and Pdgfb^cTG^ mice. We first conducted an analysis of a publicly available single-cell RNA-Seq (scRNA-Seq) data set (Gene Expression Omnibus [GEO] GSE129788), in which scRNA-Seq was performed to profile the cellular composition and transcriptomes of young and old mouse brains (41). Although many brain cell types, such as endothelial cells (ECs), VSMCs, PCs, microglia (MGs), and hemoglobin-expressing vascular cells (Hb-VCs), express *Pdgfb* in young mice, none of these cell types expressed more *Pdgfb* in aged mice (Supplemental Figure 3A). Moreover, immunofluorescence staining of brain tissue sections from Pdgfb^cTG^ mice showed no increase in the numbers of PDGF-BB–expressing MGs (TMEM119^+^ and Iba1^+^ cells) (Supplemental Figure 3, B and C) or PCs (CD13^+^ cells) (Supplemental Figure 3D). Therefore, local brain cells did not overexpress PDGF-BB in aged mice or in Pdgfb^cTG^ mice.

We then assessed whether brain calcification would occur in young Pdgfb^cTG^ mice, similar to the change observed in aged male mice. As expected, calcification lesions, as detected by Alizarin red staining, were found in the thalamus of 6-month-old Pdgfb^cTG^ mice but not in the age-matched WT mice (Figure 2E). We detected calcified nodules in 6 of 9 Pdgfb^cTG^ mice, and only 1 of 9 WT littermates had thalamic calcification (Figure 2D). OPN^+^ aggregates associated with vessels were also found in Pdgfb^cTG^ mice but not in WT mice (Figure 2F). Western blot analysis consistently showed upregulation of OPN expression in the thalamus of Pdgfb^cTG^ mice relative to WT mice (Figure 2, G and H). We also detected an increase in ALPL expression in the thalamus of young (6-month-old) Pdgfb^cTG^ mice relative to age-matched WT mice (Figure 2I).

### Normalization of circulating PDGF-BB abolishes age-associated brain calcification.

**Figure 3:**
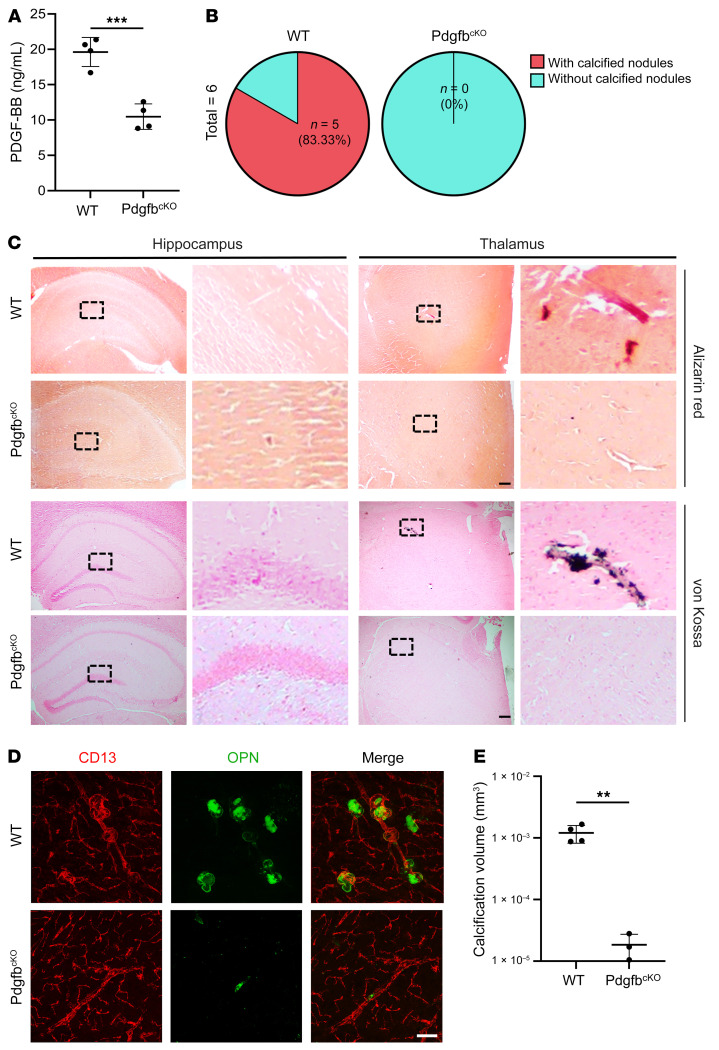
Deletion of *Pdgfb* from bone preosteoclasts alleviates brain calcification. (**A**) ELISA measurement of serum PDGF-BB concentrations in 17-month-old Pdgfb^cKO^ male mice and WT littermates. *n* = 4–5. (**B**) Calculation of the calcification incidence in Pdgfb^cKO^ mice and WT littermates based on histology. *n* = 6. (**C**) Representative images of Alizarin red– and von Kossa–stained images for 17-month-old Pdgfb^cKO^ mice and WT littermates. Calcification nodules are shown in red and black, respectively. Boxed areas are shown at higher magnification (×40) in the corresponding panels on the right. *n* = 6. (**D**) Double-immunofluorescence staining of frozen brain tissue sections from 17-month-old Pdgfb^cKO^ mice and WT littermates using antibodies against CD13 and OPN. *n* = 3–4. (**E**) Quantification of the volume of OPN^+^ calcified nodules in **D**. *n* = 3–4. The Imaris 3D reconstruction method was used to quantify the number and volume of calcification. Scale bars: 100 μm (**C** and **D**). Data are shown as the mean ± SD. **P < 0.01 and ***P < 0.001, by unpaired, 2-tailed Student’s *t* test (**A** and **E**).

We next assessed whether normalizing the level of circulating PDGF-BB using a genetic approach would attenuate cerebral vascular calcification in aged mice. We took advantage of an established conditional *Pdgfb*-KO mouse line (Pdgfb^cKO^) (4, 33, 40), in which circulating PDGF-BB levels can be reduced by approximately one-half. Consistent with the previous observation, we found that aged WT mice had increased serum PDGF-BB concentrations, whereas Pdgfb^cKO^ mice had dramatically reduced serum PDGF-BB concentrations. The reduced PDGF-BB levels were almost equivalent to those in healthy young mice (Figure 3A). While calcified nodules were present in the thalamus of aged Pdgfb^fl/fl^ (WT) mice, as detected by Alizarin red and von Kossa staining, the lesions were not detected in the same region in any of the age-matched Pdgfb^cKO^ mice tested (Figure 3, B and C). Immunofluorescence staining of brain tissue sections showed vessel-associated OPN^+^ nodules in the thalamus of aged WT mice. However, OPN^+^ nodules were not found in thalamus of Pdgfb^cKO^ mice (Figure 3, D and E). Therefore, the aged conditional *Pdgfb*-KO mice with normalized circulating PDGF-BB levels did not develop age-associated cerebral vascular calcification like the aged WT mice did.

### Recombinant PDGF-BB induces brain vessel calcification and activates the osteogenic program in an ex vivo cerebrovascular culture system.

**Figure 4:**
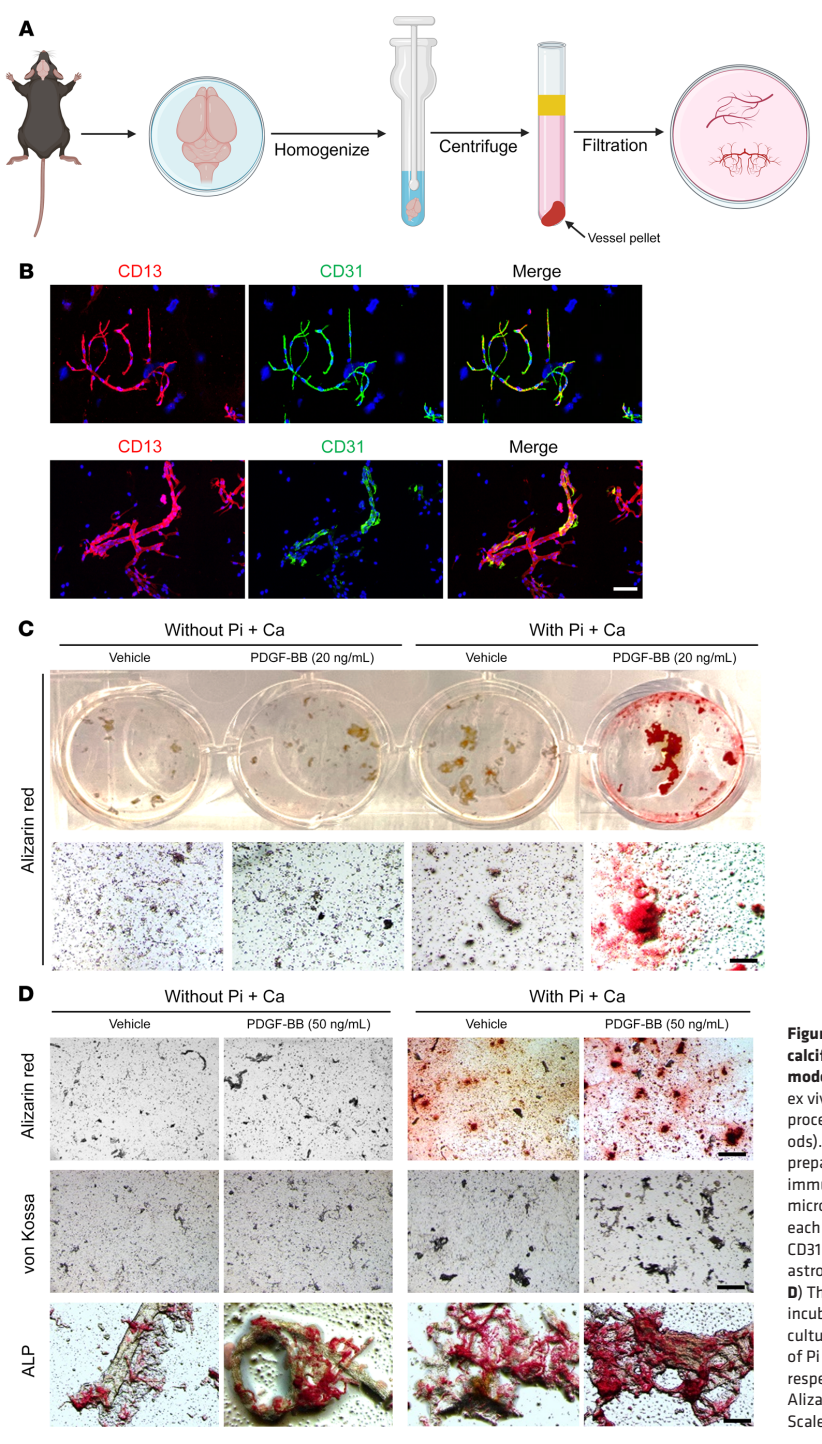
PDGF-BB treatment promotes calcification in an ex vivo cerebrovascular model. (**A**) Schematic diagram shows the ex vivo brain vessel isolation and culturing procedure (details are described in Methods). (**B**) Characterization of microvessel preparations. Representative images of immunofluorescence analysis of isolated microvessels stained with antibodies for each cell component: endothelial marker CD31 (green), PC marker CD13 (red), and astrocyte marker GFAP (green). (**C** and **D**) The isolated brain microvessels were incubated for 16 hours under the indicated culture conditions. The concentrations of Pi and Ca were 2.6 mM and 2.7 mM, respectively. Calcification was detected by Alizarin red, von Kossa, and ALPL staining. Scale bars: 100 μm.

To further characterize the molecular changes underlying cerebral vascular calcification, we developed an ex vivo cerebral microvessel culture system, in which the intact microvessel fragments isolated from the entire brain were cultured (Figure 4A). Importantly, using this method, brain vascular cell compositions, including CD31^+^ ECs, CD13^+^ PCs, and GFAP^+^ astrocyte endfeet, were consistently yielded (Figure 4B) and were able to respond to various treatments. We then incubated the cultured cerebral microvessels with recombinant mouse PDGF-BB or vehicle in the presence or absence of the mineralization inducer phosphate plus calcium (Pi+Ca). Although there were no evident mineral deposits in the microvessel incubated with Pi+Ca alone, the addition of 20 ng/mL PDGF-BB markedly increased vessel calcification, as visualized by Alizarin red staining (Figure 4C). Moreover, a higher concentration of PDGF-BB (50 ng/mL) increased more vessel calcification, as detected by Alizarin red, von Kossa, and ALPL staining (Figure 4D).

**Figure 5:**
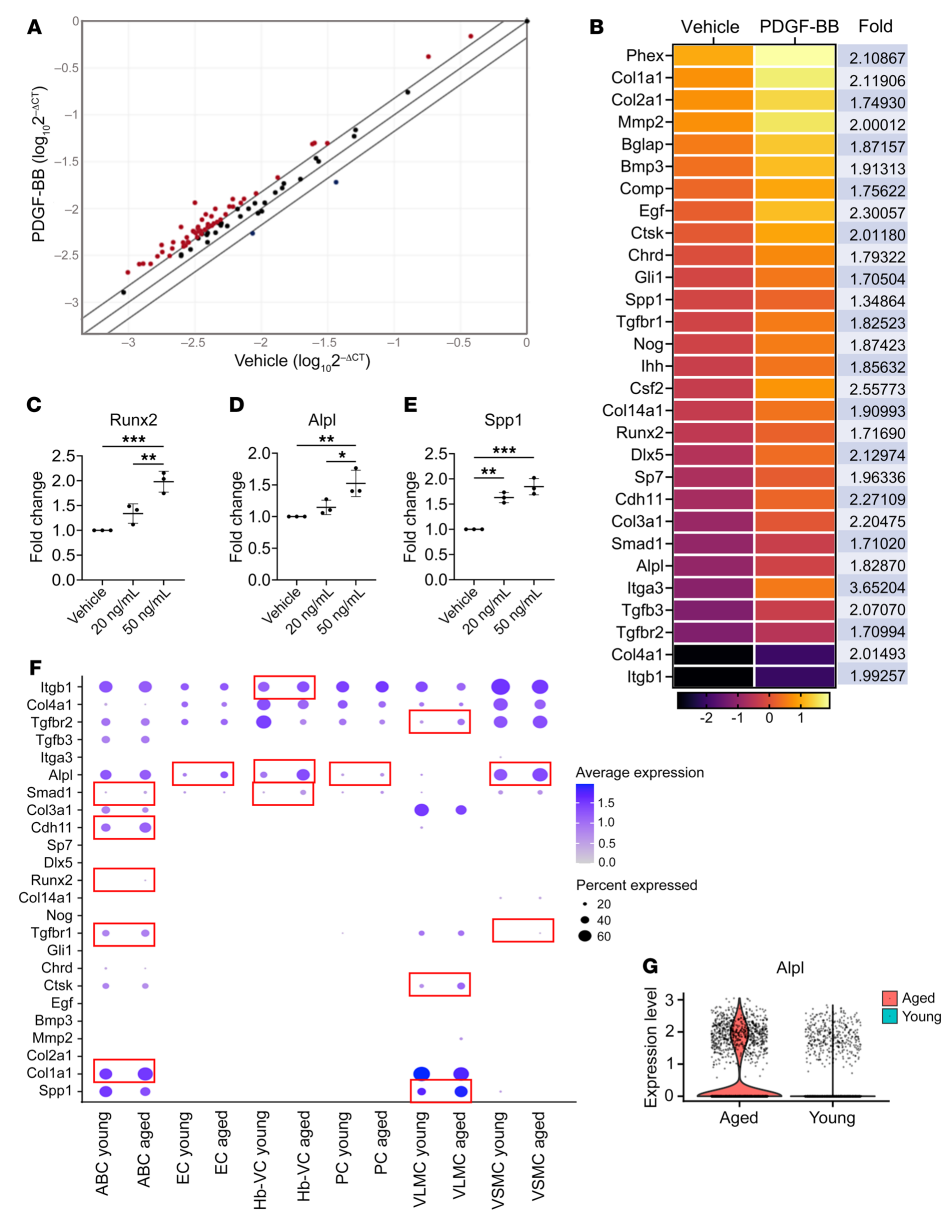
PDGFBB activates osteogenic differentiation–associated genes in the cerebral vasculature. (**A** and **B**) Osteogenic gene array analysis of cultured brain microvessels treated with or without 20 ng/mL PDGF-BB. (**A**) Expression levels of 84 osteogenesis-related genes are compared. Expression levels (2^–ΔCt^) of these genes are plotted on a logarithmic scale. The 2 boundary lines above and below the center partition line indicate the threshold of 1.5-fold upregulation and downregulation between the groups. Genes with at least 1.5-fold higher expression in PDGF-BB–treated vessels compared with vehicle-treated vessels are shown as red dots (the gene names and fold changes are listed in **B**). (**B**) Heatmap shows statistically significantly regulated genes (>1.5-fold and *P* < 0.05) in PDGF-BB–treated versus vehicle-treated microvessels. *n* = 2. (**C**–**E**) qRT-PCR analysis of *Runx2*, *Alpl*, and *Spp1* mRNA expression levels in isolated mouse brain vessels with different concentrations of PDGF-BB treatment for 16 hours. *n* = 3. (**F**) Dot plots of osteogenic gene expression in different subtypes of vascular cells from young and aged mouse brains by analysis of a scRNA-Seq data set (GEO GSE129788) (see detailed information in Methods). (**G**) Violin plot shows substantially increased expression of *Alpl* in cerebral vascular cells in the aging mouse brain. *P < 0.05, **P < 0.01, and ***P < 0.001, by ordinary 1-way ANOVA for multiple-group comparisons (**C**–**E**).

Previous studies of arterial calcification demonstrated that pathological medial calcification is a process analog to bone mineralization with VSMCs entering an osteoblast-like differentiation program with increased expression of bone-specific transcription factors and osteogenic differentiation–associated factors (42, 43). To assess whether PDGF-BB induces similar changes in cerebral microvessels, we took advantage of real-time profiler PCR array technology to chart the changes in gene expression of 84 genes related to osteogenic differentiation, including growth factors and transcription factors mediating osteogenesis. Intriguingly, the majority of osteogenesis-associated genes (80 of a total of 84 genes) were upregulated, and only 4 genes were downregulated in PDGF-BB–treated microvessels compared with vehicle-treated vessels (Figure 5A), indicating a broad activation of the osteogenic differentiation program by PDGF-BB. Following the analysis, genes that exhibited a *P* value of less than 0.05 were selected. A heatmap was then generated to visualize the expression patterns of these genes. Among the top upregulated genes, there were many bone extracellular matrix protein–encoding genes (e.g., collagen family member *Mmps*, *Bglap*, *Spp1*, and integrins), growth factor genes (*Bmps*, *Ihh*), and transcription factor genes promoting osteoblast differentiation (e.g., *Runx2*, *Sp7*, *Smad1*, *Gli1*, *Dlx5*) (Figure 5B). We further validated the gene array analysis using quantitative real-time PCR (qRT-PCR) analysis, in which PDGF-BB induced upregulation of *Runx2* (Figure 5C), *Alpl* (Figure 5D), and *Spp1* (Figure 5E) levels in cerebral microvessels. These results from the ex vivo experiments suggest that PDGF-BB promoted brain microvessel calcification by directly activating the osteogenic differentiation program. We also conducted an analysis of a publicly available scRNA-Seq data set, in which scRNA-Seq was performed to profile the cellular composition and transcriptomes of young and old mouse brains (41). We analyzed and compared transcriptional changes between young and old cell types specifically in 6 cerebral vascular cell types: arachnoid barrier cells (ABCs), ECs, PCs, VSMCs, Hb-VCs, and vascular and leptomeningeal cells (VLMCs). The genes included in our analysis are osteogenic differentiation–associated genes that were identified from our PCR array analysis as being upregulated in response to PDGF-BB. Analysis of the scRNA-Seq data set (GEO GSE129788) showed that many genes related to osteogenic differentiation were upregulated across the vascular network (Figure 5F). In particular, the violin plot showed upregulation of *Alpl* gene expression in aged versus young murine cerebral vascular cells (log fold change = 0.462, Bonferroni-adjusted *P* = 1.44 × 10^–12^; Figure 5G).

### PDGF-BB activates p-PDGFRβ/p-ERK/RUNX2 signaling in PCs to upregulate osteoblast differentiation genes.

**Figure 6:**
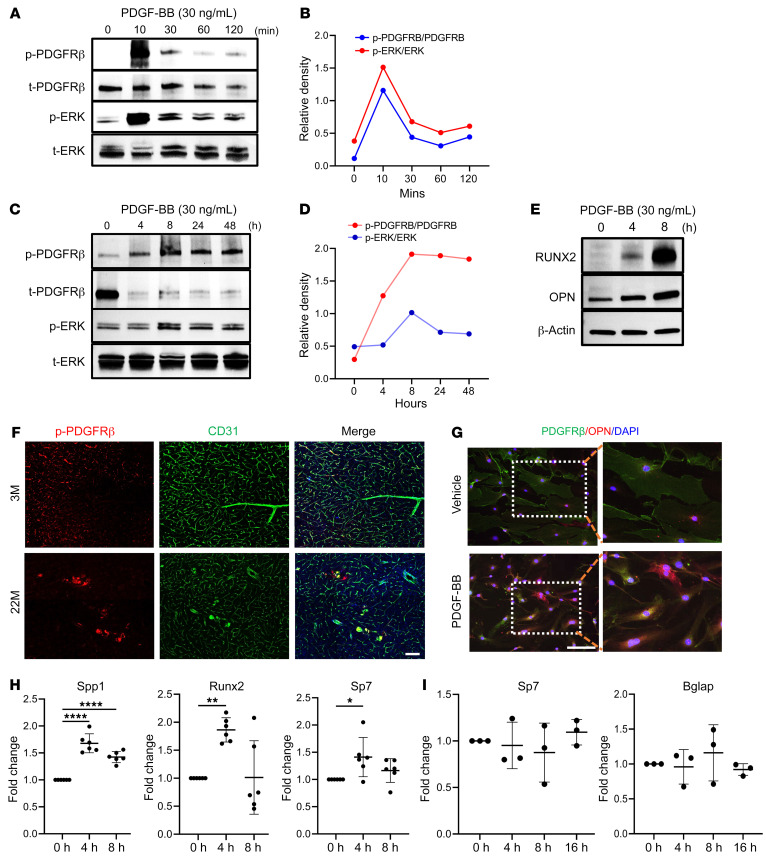
PDGF-BB stimulates p-PDGFRβ/p-ERK/RUNX2 signaling in PCs to activate multiple key osteoblast differentiation genes. (**A**–**D**) Primary mouse brain PCs were exposed to recombinant mouse PDGF-BB at a concentration of 30 ng/mL for shorter time periods (**A** and **B**) and longer time periods (**C** and **D**). Expression of the indicated proteins was detected by Western blot analysis (**A** and **C**). The relative intensities of the proteins were quantified using ImageJ (**B** and **D**). (**E**) Primary mouse brain PCs were exposed to recombinant mouse PDGF-BB at a concentration of 30 ng/mL for 4 and 8 hours. Western blot analysis of RUNX2 and OPN expression. (**F**) Double-immunofluorescence staining of frozen brain tissue sections from 3- and 22-month-old male mice using antibodies against CD31 and p-PDGFRβ. *n* = 3. (**G**) Primary mouse brain PCs were subjected to an 8-hour treatment with recombinant mouse PDGF-BB. Double immunocytochemical staining was performed using antibodies against PDGFRβ and OPN. Boxed areas are shown at a higher magnification in the corresponding panels to the right. *n* = 3. (**H**) Primary mouse brain PCs were treated with 30 ng/mL PDGF-BB for 4 and 8 hours. qRT-PCR analysis was conducted to assess the expression levels of osteogenic marker genes, including *Spp1*, *Runx2*, and *Sp7*. *n* = 6. (**I**) Primary brain astrocytes were treated with 30 ng/mL PDGF-BB for 4, 8, and 16 hours. qRT-PCR was conducted to assess the expression levels of osteogenic markers, including *Sp7* and *Bglap*. *n* = 3. Scale bars: 100 μm (**F** and **G**). Relative fold-change results are shown as the mean ± SD. *P < 0.05, **P < 0.01, and ****P < 0.0001, by ordinary 1-way ANOVA for multiple-group comparisons (**H** and **I**).

Since the blood vessel cells isolated from mouse brain in our ex vivo experiments contained a mixture of vascular cells, we assessed which cell type or types were key players in the process of calcification during aging. We first examined the changes in PDGF-BB/PDGFRβ signaling in brain PCs, which have abundant PDGFRβ expression (44–46). PCs isolated from mouse brain were treated with PDGF-BB at 30 ng/mL (equivalent serum PDGF-BB level in aged mice) for different durations. Western blot analysis showed a persistent downregulation of PDGFRβ in PDGF-BB–treated cells relative to vehicle-treated control cells (Supplemental Figure 4A). Immunocytofluorescence staining demonstrated that PDGFRβ was mostly distributed on the plasma membrane of PCs, with some protein expressed in the nucleus in the vehicle-treated control cells, whereas PDGFRβ expression on the cell membrane was markedly reduced with PDGF-BB treatment (Supplemental Figure 4B), suggesting that the PDGF-BB ligand induced downregulation of cell-surface PDGFRβ. This result is in line with our recent finding that persistent exposure of brain PCs to abnormally high concentrations of PDGF-BB leads to reduced PDGFRβ expression by inducing ectodomain shedding of PDGFRβ from PCs (32). However, despite the lowered cell-surface expression of PDGFRβ, PDGF-BB rapidly induced the activation of PDGFRβ signaling, as evidenced by increased PDGFRβ phosphorylation at Tyr751, along with the activation of downstream phosphorylated ERK (p-ERK) (Figure 6, A and B). p-PDGFRβ and p-ERK were maintained at higher levels in longer-term PDGF-BB–treated cells compared with those without PDGF-BB treatment (Supplemental Figure 4B and Figure 6, C and D). Moreover, PDGF-BB treatment markedly upregulated the further downstream effectors RUNX2 and OPN (Figure 6E). The results suggest that even when cell-surface PDGFRβ was reduced in PCs due to persistent high levels of PDGF-BB during aging, the remaining low level of PDGFRβ was still sufficient to trigger robust activation of the downstream p-ERK/RUNX2 signaling pathway. Increased p-PDGFRβ and OPN expression in PCs was also confirmed by immunofluorescence staining of brain tissue sections from aged mice (Figure 6F) and cytofluorescence staining of isolated mouse brain PCs with PDGF-BB treatment (Figure 6G). Moreover, qRT-PCR results showed greatly increased expression of *Runx2* in PDGF-BB–treated PCs relative to vehicle-treated control cells (Figure 6H). *Sp7* and *Spp1*, direct *Runx2* downstream targets and key osteoblast differentiation genes, were also upregulated in cells with PDGF-BB treatment. As PDGFRβ is also expressed in astrocytes, we assessed whether osteoblast differentiation genes were stimulated in astrocytes by PDGF-BB treatment at the same concentration used in the PC culture. We observed no differences in mRNA expression of *Osx* or *Bglap* in PDGF-BB–treated compared with vehicle-treated astrocytes at various time points (Figure 6I), indicating transdifferentiation of PCs but not astrocytes toward osteoblast-like lineage cells.

### PDGF-BB activates the phosphate transporter Slc20a1 in astrocytes.

**Figure 7:**
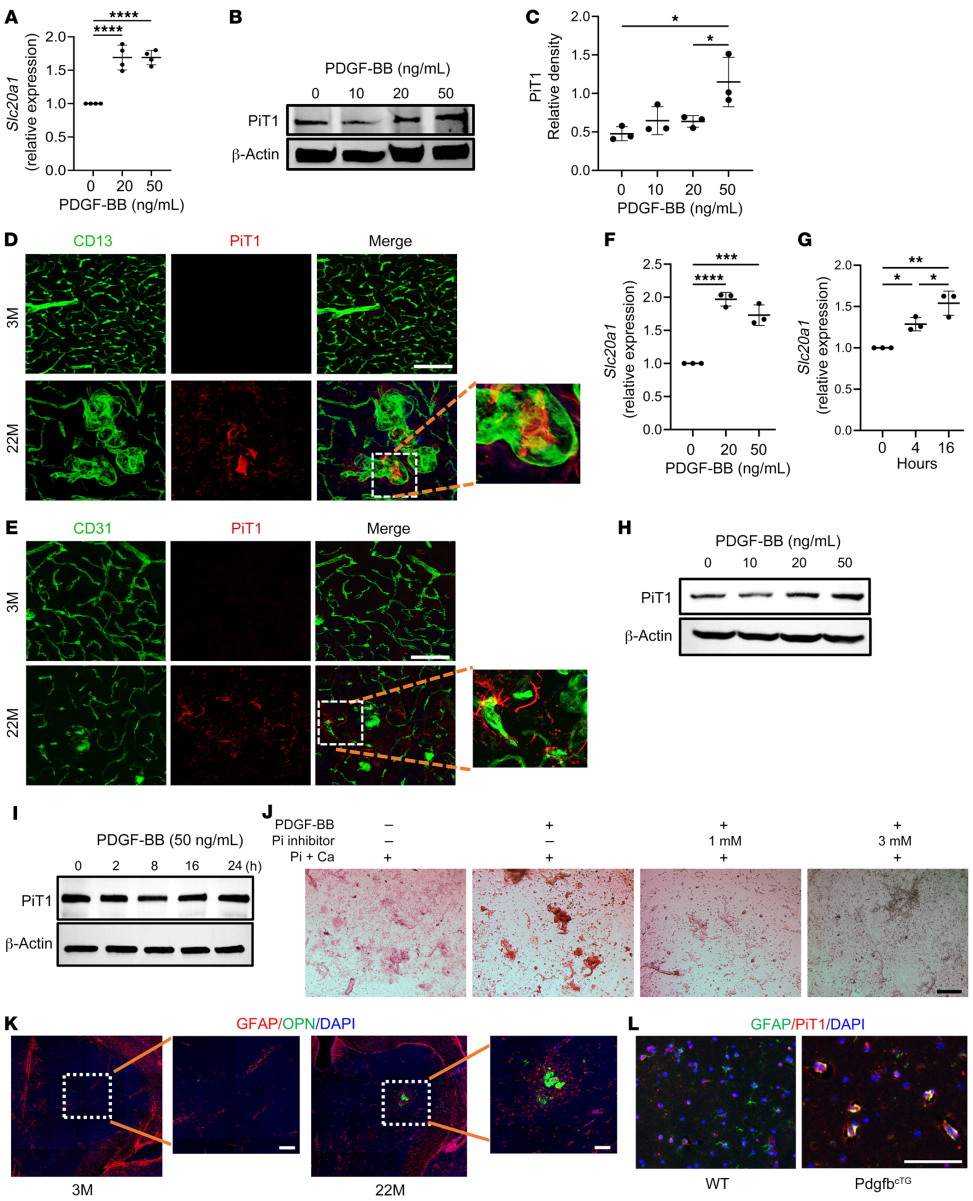
PDGF-BB upregulates phosphate transporters PiT1 (*Slc20a1*) in astrocytes. (**A**) qRT-PCR analysis of *Slc20a1* mRNA expression in isolated mouse brain microvessels with different dosages of PDGFBB treatment for 16 hours. *n* = 4. (**B**) Western blot analysis of PiT1 protein expression in isolated mouse brain microvessels with different dosages of PDGF-BB treatment for 16 hours. *n* = 3. (**C**) The relative density of PiT1 was calculated using ImageJ. *n* = 3. (**D**) Double-immunofluorescence staining of frozen brain tissue sections from 3- and 22-month-old male mice using antibodies against CD13 and PiT1. *n* = 3. (**E**) Double-immunofluorescence staining of frozen brain tissue sections from 3- and 22-month-old male mice using antibodies against CD31 and PiT1. *n* = 3. (**F** and **G**) qRT-PCR analysis of *Slc20a1* mRNA expression in primary brain astrocytes treated with PDGF-BB at different dosages (**F**) and for different durations (**G**). *n* = 3. (**H**) Western blot analysis of PiT1 protein expression in brain astrocytes treated with PDGF-BB at different dosages. (**I**) Western blot analysis of PiT1 protein expression in brain PCs treated with 50 ng/mL PDGF-BB at different time points. (**J**) Isolated brain microvessels were incubated under different culture conditions indicated for 16 hours with 2.6 mM Pi, 2.7 mM Ca, and 20 ng/mL PDGF-BB. The concentrations of the Pi transporter inhibitor PFA were 1 mM and 3 mM. Calcification was detected by Alizarin red staining. (**K**) Double-immunofluorescence staining of frozen brain tissue sections from 3- and 22-month-old male mice using antibodies against GFAP and OPN. *n* = 3. (**L**) Double-immunofluorescence staining of frozen brain tissue sections from WT and Pdgfb^cTG^ mice using antibodies against GFAP and PiT1. *n* = 3. Scale bars: 100 μm (**J**–**L**); 200 μm (**D** and **E**). Relative fold-change results are shown as the mean ± SD. *P < 0.05, **P < 0.01, ***P < 0.001, and ****P < 0.0001, by ordinary 1-way ANOVA for multiple-group comparisons (**A**, **C**, **F** and **G**). Magnification value is ×10 for **D**, **E**, and **K**.

It has been reported that PDGF-BB increases the expression of *Slc20a1*, a type III sodium-dependent Pi transporter, in aortic SMCs (47, 48). *Slc20a1* is distributed in many CNS cell types, including astrocytes, neurons, and ependymocytes (41). We then tested whether PDGF-BB also activates *Slc20a1* expression in cerebral microvessels, as PiT1 (*Slc20a1*-encoded protein) is a key regulator of skeletal mineralization. As we expected, recombinant mouse PDGF-BB dose-dependently increased the mRNA expression of *Slc20a1* in the ex vivo–cultured cerebral vessels (Figure 7A). Consistently, Western blot analysis also showed that PiT1 expression was elevated by PDGF-BB treatment in a dose-dependent manner (Figure 7, B and C). The PDGF-BB failed to increase *Slc20a2* and *Xpr1* expression in our ex vivo microvessel assays (Supplemental Figure 5, A and B). We assessed PiT1 expression in the thalamus of aged male mice. Double-immunofluorescence staining of brain tissue sections demonstrated that PiT1 expression was undetectable in 3-month-old young mice, whereas a PiT1^+^ signal was detected within the calcified lesions that were associated with CD13^+^ and CD31^+^ brain capillaries in 22-month-old mice (Figure 7, D and E). We also tested whether PiT1 is required for PDGF-BB–induced brain vessel calcification by adding phosphonoformic acid (PFA), a specific inhibitor of phosphate transporter, in the PDGF-BB–treated ex vivo cerebral microvessels (49, 50). While PDGF-BB stimulated calcification of the microvessels, adding low (1 mM) and high (3 mM) dosages of PFA efficiently blocked the effect (Figure 7J). The result suggests that PiT1 mediates PDGF-BB–induced cerebral vascular calcification. We further characterized the specific cell type(s) within the cerebral vasculature that are responsible for PDGF-BB–induced *Slc20a1* expression. We postulated that PCs and/or astrocytes might be the targets, as both cell types have abundant PDGFRβ expression. Our results showed that PDGF-BB stimulated the upregulation of *Slc20a1* gene expression in astrocytes in a dose- and time-dependent manner (Figure 7, F and G). Consistently, PDGF-BB at high concentration induced upregulation of PiT1 protein expression in astrocytes as detected by Western blot analysis (Figure 7H), whereas it failed to exert the same effect on PCs (Figure 7I). Furthermore, GFAP^+^ astrocytes accumulated surrounding the OPN^+^ calcified nodules (Figure 7K). Importantly, while a PiT1^+^ signal was not detected in the thalamic astrocytes of WT control mice, many GFAP^+^ astrocytes exhibited PiT1 expression in Pdgfb^cTG^ mice (Figure 7L), providing in vivo evidence for the involvement of astrocytes in the mineralization process of brain calcification.

**Figure 8:**
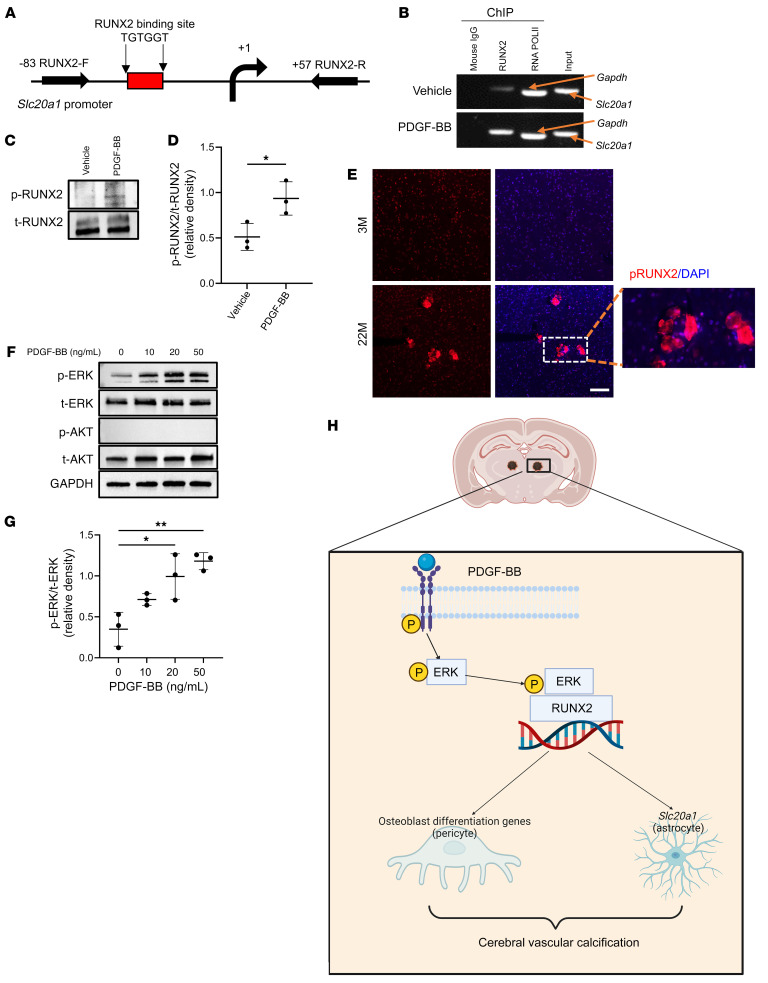
PDGF-BB activates *Slc20a1* gene transcription through ERK/RUNX2 signaling. (**A**) Schematic representation of the *Slc20a1* promoter region. The location of the consensus RUNX2 binding site (TGTGGT) and the regions chosen for PCR amplification by the primers in the ChIP-qPCR assays are indicated. (**B**) Cerebral microvessels isolated from brain were treated with 20 ng/mL PDGF-BB or vehicle. Chromating DNA was immunoprecipitated using a specific antibody against RUNX2 or mouse IgG (negative control). DNA fragments were amplified with primers specific for the *Slc20a1* promoter. As a positive control, an antibody against RNA polymerase II (RNA PolII) was used for immunoprecipitation, and primers specific for GAPDH were used for PCR. (**C**) Cerebral microvessels isolated from brain were treated with 20 ng/mL PDGF-BB or vehicle. Western blot analysis of p-RUNX2 and total RUNX2 (t-RUNX2). *n* = 3. (**D**) The relative density of p-RUNX2 to t-RUNX2 was calculated using ImageJ. *n* = 3. (**E**) Immunofluorescence staining of frozen brain tissue sections from 3- and 22-month-old male mice using antibodies against RUNX2. *n* = 3. Scale bar: 100 μm. Magnification ×10. (**F**) Cerebral microvessels isolated from brain were treated with increasing concentrations of PDGF-BB. Western blot analysis of p-ERK and p-AKT. *n* = 3. (**G**) Relative densities of p-ERK and t-ERK were calculated using ImageJ (*n* = 3). (**H**) Schematic model showing the molecular mechanisms underlying PDGF-BB–induced brain vascular calcification. All data are shown as the mean ± SD. *P < 0.05 and **P < 0.01, by ordinary 1-way ANOVA for multiple-group comparisons (**G**). *P < 0.05, by unpaired, 2-tailed Student’s *t* test for 2-group comparisons (**D**).

We next attempted to gain mechanistic insights into PDGF-BB–induced *Slc20a1* expression in cerebral vessels. *Runx2*, the master organizer in controlling the osteoblast differentiation program (51), was among one of the top genes that differentially changed in PDGF-BB–treated versus vehicle-treated cerebral microvessels in the osteogenesis PCR array (Figure 5, B and C). It is noteworthy that a consensus RUNX2 binding site (TGTGGT) (52) was found in *Slc20a1* promoter regions, nearby the transcription start site (Figure 8A). We then used the ChIP assay to test whether RUNX2 could bind to the *Slc20a1* promoter in cerebral microvessels in response to PDGF-BB treatment. Chromatin from the PDGF-BB– and vehicle-treated cerebral microvessels was immunoprecipitated using a specific antibody against RUNX2. Genomic DNA fragments bound to RUNX2 were analyzed by PCR using random primers designed to include the putative RUNX2 binding sites in the *Slc20a1* promoter region (Figure 8A). No genomic DNA was pulled out in the immunoprecipitates from normal mouse IgG (Figure 8B, lane 1). As a positive control, RNA PolII antibody efficiently pulled out DNA of *GAPDH* (Figure 8B, lane 3). Importantly, there was only a weak binding of RUNX2 at the *Slc20a1* promoter in vehicle-treated cerebral vessels (Figure 8B, lane 2 in upper panel), and the binding was dramatically enhanced in the PDGF-BB treatment group (Figure 8B, lane 2 in lower panel). Therefore, RUNX2 indeed bound the *Slc20a1* promoter region for its gene transcription. Accumulating evidence demonstrated that RUNX2 is a target of the kinases MAPK, ERK, and AKT, which induce RUNX2 protein phosphorylation to enhance its activity (53–58). Indeed, we observed that PDGF-BB induced upregulation of p-RUNX2 (Figure 8C, upper panel, and Figure 8D). Consistently, immunofluorescence staining of brain tissue sections showed strong p-RUNX2^+^ signal at the calcified lesions in the thalamus of aged mice but not in that of young mice (Figure 8E). It has been recognized that in VSMCs, PDGF-BB can activate ERK MAPKs (59, 60) and PI3K/Akt signaling (61, 62), which may induce RUNX2 phosphorylation. We then tested this assumption in the ex vivo cerebral vessel culture system. Indeed, we found that recombinant PDGF-BB treatment greatly enhanced the phosphorylation of ERK in a dose-dependent manner compared with the vehicle-treated group, whereas the phosphorylation of Akt was not stimulated by PDGFF-BB (Figure 8, F and G). Taken together, these findings indicate that elevated PDGF-BB in a pathological context bound to its receptor in vascular cells, leading to a robust activation of the p-PDGFRβ/p-ERK/RUNX2 signaling cascade. As a transcription factor, RUNX2 played a dual role. First, it activated the transcription of downstream osteogenic genes in PCs, promoting their transdifferentiation into osteoblast-like cells. Second, it directly stimulated gene transcription of the phosphate transporter *Slc20a1* in astrocytes, thereby leading to an impaired phosphate balance to promote mineralization of the brain vasculature (Figure 8H).

## Discussion

The skeletal system serves not only as a passive structure providing physical support and facilitating movement but also functions as an endocrine organ. Cells within the bone, including osteoblasts, osteoclast lineage cells, and bone marrow adipocytes, are capable of secreting molecules that regulate various physiological processes (31, 63, 64). We previously demonstrated that preosteoclasts, which are precursor cells of osteoclasts, secrete a much higher amount of PDGF-BB, making it a primary contributor to the elevated levels of circulating PDGF-BB in aged mice compared with young mice (4, 32). Importantly, bone-derived PDGF-BB is responsible for mediating age-associated changes, such as arterial stiffening in large arteries (4) and blood-brain barrier (BBB) impairment in hippocampal microvessels (32). Here, we provide evidence that bone-derived PDGF-BB also contributed to the development of age-associated brain calcification in male mice. Our results show that the young conditional *Pdgfb*-transgenic (Pdgfb^cTG^) mice, in which the serum PDGF-BB concentration was abnormally high, developed calcified nodules in the thalamus similar to those seen in aged mice. Moreover, age-dependent thalamic vascular calcification did not occur in Pdgfb^cKO^ mice, in which serum PDGF-BB levels were normalized to those in young WT mice. Therefore, PDGF-BB secreted by preosteoclasts was both necessary and sufficient for the development of vascular calcification in the thalamus of aging male mice, thereby reinforcing the interrelationship between bone and the vascular system. Our previous work provided evidence that elevated circulating PDGF-BB levels in aged mice or in Pdgfb^cTG^ mice are mainly generated by preosteoclasts in the bone and bone marrow, rather than being derived from myeloid cells in the blood (4). In this study, we show that brain myeloid cells in Pdgfb^cTG^ mice did not produce more PDGF-BB than their WT littermates. This result further confirms that the elevated circulating PDGF-BB in transgenic mice was primarily produced by preosteoclasts in the skeletal system and was not a local effect of cells within the brain. Thus, the present study establishes the role of bone-derived PDGF-BB as a key contributor to age-associated brain calcification, providing evidence for an intriguing interplay between the bone and the brain, which has recently been gaining attention (65, 66).

It is well accepted, from blood exchange experiments in heterochronic parabionts and heterochronic plasma transfer, that circulating pro-aging factors contribute to the acceleration of age-related brain changes (32, 67). Our data from the present study suggest that PDGF-BB is a systemic pro-aging factor that mediates age-associated brain calcification. Physiological levels of PDGF-BB/PDGFRβ are essential to maintain cerebrovascular homeostasis in healthy young mice, and global *Pdgfb*- or *Pdgfrb*-KO mice (*Pdgfb^–/–^* or *Pdgfrb^–/–^)* are perinatally lethal (68, 69). Our study shows that abnormally high PDGF-BB levels during aging were associated with brain calcification, suggesting that an excess of PDGF-BB also leads to pathological brain changes. Therefore, a fine balance of PDGF-BB levels is important for the maintenance of physiological functions. The mouse models used in this study had preosteoclast-specific *Pdgfb* deletion or overexpression. It will be of interest in the future to determine whether brain calcification in aged male mice could also be inhibited by deleting 1 allele of *Pdgfb* or *Pdgfrb* using heterozygously *Pdgfb*- or *Pdgfrb*-deficient mice (*Pdgfb^+/–^* or *Pdgfrb^+/–^*).

Intracranial vessel–associated calcifications are commonly found in elderly individuals and in those with neurodegenerative diseases, type I interferonopathies, brain tumors, or other disorders (13, 14). Brain vascular calcification in animals has not been systemically characterized, and the pathogenic mechanisms are largely unexplored. In the present study, we visualized and quantified brain calcifications in aged mice using several approaches: whole-brain MRI sequence of SWI with phase-mapping and μCT scanning, Alizarin red staining, von Kossa staining, and immunofluorescence staining of brain tissue sections, as well as ex vivo cerebral microvessel culturing. We demonstrate that calcified nodules were associated with brain capillaries and were primarily located in the thalamic region in aged male mice. Surprisingly, we observed calcified nodules in the thalamic region in 12 of 15 male mice at the age of 22 months, but none of the 9 female mice of the same age developed brain calcification, as confirmed by both SWI of whole brains and Alizarin red staining of brain tissue sections. Thus, there was a clear sex difference in brain calcification development with age in mice. This finding is supported by a large cohort study involving 11,941 individuals, which revealed a higher frequency of choroid plexus calcification, the most common form of brain calcification in aged (>50 years) individuals, in males than in females (70). Additionally, our results demonstrate a sex difference with regard to changes in serum PDGF-BB levels with age. Contrary to the higher serum PDGF-BB concentration observed in aged male mice, the serum PDGF-BB concentration in aged female mice was lower compared with that in young female mice. This finding aligns with a human study in which female individuals had decreased serum PDGF-BB levels with advancing age (71). The differential alterations in PDGF-BB levels with age between male and female mice likely contributed to the sex difference in the rate of brain vascular calcification.

Our results from ex vivo microvessel culture and in vitro cell culture studies uncovered the p-PDGFR/p-ERK/RUNX2 signaling cascade as a key mediator for elevated PDGF-BB–induced cerebrovascular calcification. Our ex vivo cerebral vascular culture model was developed on the basis of previous publications (72, 73) and then modified. This approach enabled the isolation of high-purity and high-integrity microvessel fragments of consistent cell composition and was therefore an ideal approach to explore molecular mechanisms underlying PDGF-BB–induced vascular calcification in the brain. Using this system, we found that PDGF-BB treatment activated ERK, which in turn upregulated RUNX2. RUNX2 is a master gene regulator of skeletogenesis, as it tunes the mesenchymal stem cell commitment toward the osteoblast lineage. It is known that RUNX2, as a transcription factor, binds the promoter region of many downstream targets (74). In particular, RUNX2 activation in mesenchymal stem cells stimulates expression of the earlier osteoblastic markers *SP7* (osterix-encoding gene), *Alpl* (ALP-encoding gene), *Col1a* (collagen type Iα1–encoding gene), and, later, *Spp1* (OPN-encoding gene) and *Bglap* (OCN-encoding gene) (75). The promoter regions of all of these genes have a specific sequence for the RUNX2 DNA–binding site (76, 77). Importantly, in our PCR array of PDGF-BB–treated ex vivo cerebral microvessels, almost all of these genes were upregulated. scRNA-Seq analysis of differentially expressed genes in aged versus young vascular cells from whole brain also identified changes in several of these genes. Our results are in agreement with the findings from *Pdgfb^ret/ret^* mice, which demonstrated that the cells surrounding calcified nodules express markers for osteoblasts, osteoclasts, and osteocytes (21, 28, 35, 38, 68). Our in vitro cell culture assays further demonstrated that PCs and astrocytes were the major cell types involved in the vascular calcification process. On one hand, RUNX2 activation in PCs upregulated multiple osteoblast differentiation–associated genes in this specific vascular cell type. PCs are widely believed to function as mesenchymal stem cells that can differentiate into multiple cell types (78–83). Our results suggests that a persistently high level of PDGF-BB stimulation activated key osteoblast differentiation genes in PCs for their transdifferentiation into osteoblast-like cells. On the other hand, RUNX2 in astrocytes directly activated gene transcription of the phosphate transporter PiT1, which encodes the *Slc20a1* gene, thereby facilitating the pathological accumulation of Pi and tissue mineralization. It has been reported that reactive astrocytes are always present around brain calcification and may be an important player in the development of PFBC (35). Our results consistently showed the accumulation of GFAP^+^ astrocytes surrounding the calcified nodules, indicating the involvement of this cell type in brain calcification. Taken together, we propose that the 2 synergistic processes associated with 2 different vascular cell types (e.g., PCs and astrocytes) contribute to the development of calcification in thalamic microvessels associated with aging (Figure 8G). Of note, upregulation of the key components in the p-PDGFR/p-ERK/RUNX2 signaling pathway as well as the downstream targets was also validated in the brain tissue of aged mice and conditional *Pdgfb*-transgenic mice.

We recently demonstrated that increased serum PDGF-BB concentrations in aged male mice induces a loss of PDGFRβ expression in PCs and compromises BBB permeability specifically in the hippocampus (32), whereas the present study showed an increase in the activated form of PDGFRβ (p-PDGFRβ) and its further downstream RUNX2 signaling activation exclusively in the thalamus. The findings suggest that, while aberrantly elevated PDGF-BB downregulate PDGFRβ in hippocampal PCs, it activates the p-PDGFRβ/p-ERK/RUNX2 signaling cascade specifically in thalamic PCs. The region-specific effect of PDGFB/PDGFRβ signaling is likely attributable to the distinct distribution and activity of vascular cells in different brain regions. Indeed, recent studies using mouse and human scRNA-Seq have revealed the diversity and region-specific patterns of brain vascular cells, including astrocytes and PCs, highlighting the regional heterogeneity of the cerebral vasculature (32, 84–87). Notably, human brain regions have been found to contain 2 subclusters of PCs, known as M-PCs associated with extracellular matrix changes and T-PCs involved in transport (85). In our analysis of a publicly available data set (GEO GSE116470) (88), we observed a substantial disparity in the distribution of M-PCs and T-PCs among different brain regions. M-PCs were more abundant in the hippocampus than in the thalamus, whereas T-PCs were more enriched in the thalamus than in the hippocampus (data not shown). These findings suggest that the distinct patterns of PC subtypes across various brain regions, along with their distinct functional properties, may contribute to the region-specific changes observed during aging.

In patients with PFBC and mouse models (e.g., *Pdgfb^ret/ret^*), there are bilateral brain vessel calcifications (21, 28, 35, 38). Previous in vitro studies provided evidence from a cell culture study that PDGFB or PDGFRB mutations identified in PFBC are mostly loss of function (68, 89). Specifically, these mutations resulted in declined protein expression or activity of PDGFRβ in the cells transfected with most of the individual mutants. Our data show that persistently high concentration of PDGF-BB ligand during aging downregulated PDGFRβ, but the remaining lower level of PDGFRβ was still sufficient to trigger robust activation of the downstream p-ERK/RUNX2 signaling cascade. To our knowledge, there have been no direct measurements of PDGFRβ signaling levels using brain samples from patients with PFBC and from *Pdgfb^ret/ret^* mice that developed brain calcifications. Based on our findings in aged mice, it is likely that PDGFRβ downstream signaling, such as p-ERK/RUNX2, is more or less activated in PCs with these PDGFB or PDGFRB mutations. Additional evidence to support this assumption comes from previous genetic mouse studies. For example, *Pdgfb^ret/ret^* mice, in which PDGF-BB exhibits a partial loss of function due to C-terminal truncation but retains its ability to bind to and activate PDGFRβ, develop severe brain calcification (28). However, *Pdgfb* and *Pdgfrb* double-heterozygous-KO mice and *Pdgfrb^redeye/redeye^* mice with nearly abolished PDGF-BB/PDGFRβ signaling do not develop brain calcifications (68). These findings suggest that PDGFRβ may have a certain threshold for the activation of downstream signaling in PCs. If the level of PDGFRβ is higher than the threshold, downstream p-ERK/RUNX2 signaling can still be activated to trigger calcification; if it is lower than the threshold, brain calcification will not occur. Human studies evaluating the correlation between brain calcification and PDGFRβ signaling in brain tissue from individuals with PFBC and aged individuals are lacking. Further investigation into the changes in PDGFRβ signaling, especially p-ERK/RUNX2, cross-sectionally in brain samples from patients with PFBC and elderly individuals is crucial for a better understanding of species differences and regional differences in brain calcification and the development of targeted treatment strategies for the disorder.

## Methods

### Mouse line.

Male mice at the age of 3, 6, 17, 21, and 22 months and female mice at the age 3 and 22 months were used in this study. The following mouse strains were used: C57BL6/J (National Institute on Aging); *Trap-Cre* (provided by Jolene J. Windle, Virginia Commonwealth University, Richmond, Virginia, USA); *Pdgfb^fl/fl^* (The Jackson Laboratory, 017622); *Trap-Cre* *Pdgfb^fl/fl^* (referred to herein as Pdgfb^cKO^ mice, generated by breeding *Trap-Cre* and *Pdgfb^fl/fl^* mice); and Trap-Pdgfb^cTG^ (generated in our laboratory and described in refs. 4, 40). Mice were housed in a well-maintained, temperature-controlled environment with a consistent 14-hour light/10-hour dark cycle and 67°F–77°F and 30%–70% relative humidity, which aligned with standard animal housing guidelines. We performed PCR studies of genomic DNA extracted from mouse tails to determine the genotype of the mice (4, 40).

### SWI MRI studies.

SWI MRI was performed on an 11.7 T Biospec system (Bruker Ettlingen) equipped with a horizontal bore and an actively shielded pulse-field gradient (maximum at 0.74 T/m). Operation procedures followed those used in previous studies (90, 91). Briefly, anesthesia of mice was induced using 1.5% isoflurane in medical air (78% N_2_ plus 21% O_2_) for 15 minutes and maintained throughout the experiment using 1.25% isoflurane. At approximately the tenth minute after anesthetic induction, the mice were relocated to a water-heated animal bed with temperature control and positioned with a bite bar and ear pins. Images were acquired using a 72 mm quadrature volume resonator as a transmitter and a 4-element (2 × 2), phased-array coil as a receiver. The homogeneity of the B_0_ (intensity of static magnetic field of MRI scanner) field over the mouse brain was improved with a global shimming (up to the second order) based on a subject-specific preacquired fieldmap. A multislice, fast low-angle shot (FLASH) sequence was used with the following parameters: TR/TE = 900/3.0 ms, flip angle = 30°, average number = 4, field of view (FOV) = 15 × 15 mm^2^, matrix size = 256 × 256, spatial resolution = 59 × 59 μm^2^, slice number = 16, slice thickness = 0.75 mm, partial Fourier acquisition factor (phase-encoding direction) = 0.75, and scan duration = 11.5 minutes.

### μCT scanning.

We performed μCT scanning using the same method as in a previous study (28). Mice were anesthetized using isoflurane and then sacrificed. The brain tissues were fixed in 4% paraformaldehyde at 4°C for 24 hours and incubated in phosphotungstic acid to increase the soft-tissue contrast. High-resolution μCT (Skyscan 1275, Bruker MicroCT) (voltage, 58 kVp; current, 172 uA; resolution, 8 μm/pixel) was used for scanning. The images were reconstructed with NRecon image reconstruction software, version 1.6 (Bruker MicroCT).

### Serum collection and ELISA analysis of PDGF-BB concentrations.

Approximately 0.5 mL blood was collected from the right ventricle of the mice. Blood was transferred into an Eppendorf tube and left undisturbed for 2 hours, followed by centrifugation to collect the serum. The Mouse/Rat PDGF-BB Quantikine ELISA Kit (MBB00, R&D Systems) was used to determine the amount of PDGF-BB. Concentrations were assessed at 450 and 570 nm using a spectrophotometer (A51119600DPC, Thermo Fisher Scientific).

### Mouse brain tissue processing, immunofluorescence staining, and histochemistry.

Mice were perfused using PBS followed by phosphate-buffered 4% paraformaldehyde. Brains were collected, fixed in phosphate-buffered 4% paraformaldehyde for 24 hours at 4°C, and separated sagittally. Paraffin-embedded and frozen brain tissue sections were prepared as described previously (32). For immunofluorescence staining, 50 μm thick brain tissue sections were incubated with primary antibodies for 3 days at 4°C before the blocking and antigen retrieval procedure. The sections were then incubated overnight with the corresponding fluorescence-linked secondary antibodies (Jackson ImmunoResearch Laboratories). Images were obtained using confocal microscopes (Zeiss LSM780 FCS, Leica SP5) and analyzed with the image processing software Imaris 9.2.0. (Bitplane) (35) and ImageJ (NIH). The following primary antibodies were used: CD13 (1: 200, MCA2183, Bio-Rad); OPN (1:200, AF808, R&D Systems); CD31 (1:200, FAB3628G, R&D Systems); PiT1 (1:200, 12423-1-AP, Proteintech); PiT1 (1:200, GTX64727, GeneTex); OCN (1:200, 23418-1-AP, Proteintech); ALPL (1:100, AF2910, R&D Systems); p-RUNX2(1:100, PA5-105642, Thermo Fisher Scientific); PDGFRβ (1:200, AF1042, R&D Systems); PDGFRβ (1:200, 3169S, Cell Signaling Technology); p-PDGFRB (1:200, 3161S, Cell Signaling Technology); CatK (1:200, ab19027, Abcam); and GFAP (1:500, NBP-05198, Novus Biological). Alizarin red (IW-3001, IHC World) and von Kossa staining (IW3014, IHC World) were performed using paraffin-embedded samples and following the same procedure described by the manufacturer. Quantification of the calcified nodule volume in the immunofluorescence-stained images was conducted using a previously described approach (35). Briefly, OPN-immunostained images were captured using a confocal microscope (Zeiss LSM780 FCS, Leica SP5). Imaris software was used to acquire 3D views, and image reconstruction was conducted. The volume of the OPN^+^ calcified nodules was then calculated.

### Mouse bone tissue processing and immunofluorescence staining.

Processing of femoral bone tissue and subsequent immunofluorescence staining of the bone tissue sections were conducted using the same methods described in our previous publications (4, 92, 93). Briefly, the femur tissues were harvested, fixed, decalcified, dehydrated, and embedded in OCT compound and frozen at –80°C. Coronal sections with a thickness of 10 μm were used for immunofluorescence analysis using the antibody specific for PDGF-BB (1:200, ab23914, Abcam).

### Mouse brain vessel isolation, ex vivo vessel culture, and immunofluorescence staining.

The brain vessels were isolated using methods described in previous publications (72, 73) with modification. Briefly, the entire brain was collected from mice, and the olfactory bulb and cerebellum were removed. The brain tissue was homogenized by aspirating and pushing out tissue using 21 gauge cannulas. The homogenates were centrifuged at 2,000*g* for 10 minutes at 4°C. After discarding the supernatant, 3 mL ice-cold dextran solution in HBSS was added, and the tube was shaken violently for 1 minute. When there was no deposit on the bottom, the tube was centrifuged at 4,400*g* for 15 minutes at 4°C. After centrifugation, the top layer was discarded, and the vessel pellet was suspended. The vessel fragments were separated using a 20 μm mesh filter (NY2002500, MilliporeSigma) and washed. For ex vivo brain vessel culturing, the pellet was carefully resuspended in EGM-2 Endothelial Cell Growth Medium (CC-3162, Lonza) with an antibiotic. The vessel fragments were transferred into a culture well. After 24 hours, the medium was changed and incubated with different treatments: recombinant mouse PDGF-BB (78178, STEMCELL Technologies), 2.7 mM calcium chloride, and 2.6 mM inorganic phosphate (NaH_2_PO_4_). Isolated brain vessel fragments were attached to slides for immunofluorescence staining. After fixation, the slides were washed with PBS and blocked by 1% BSA and 0.1% Triton X-100 overnight in PBS. All slides were then incubated with a primary antibody overnight and then a secondary antibody for 4 hours at 4°C.

### Primary cell isolation.

Pericytes were isolated from 3-month-old WT C57BL6/J mice, following the protocol described in a previous publication (94). Briefly, the brain was dissociated, cut into small pieces, and subsequently centrifuged at 290*g* for 5 minutes to remove the supernatant. The brain sample was then dissociated using the papain dissociation system (LK003176, Worthington Biochemical Corp.) for 70 minutes at 37°C, and the solution was homogenized using a 21 gauge needle. Following trituration, the myelin was separated by centrifugation at 1,360*g* for 10 minutes using a 22% BSA solution. After removal of the myelin, the blood pellet was washed with EC growth medium (ECGM) (CC-3162, Lonza). The cell pellet was then resuspended and seeded onto a 6-well plate. During the first through third passages, the PCs were cultured using ECGM (CC-3162, Lonza). Subsequently, PC growth medium (1201, ScienCell) was used to culture the cells for the remaining passages. After 6 passages, immunocytochemistry was performed to assess the purity of the isolated cells. Primary brain astrocytes (catalog 1800) were purchased from ScienCell.

### Immunocytochemistry.

After treatment, cells were fixed and incubated overnight at 4°C with specific primary antibodies targeting PDGFRβ (1:200, 3169S, Cell Signaling Technology) and OPN (1:200, AF808, R&D Systems). Subsequently, the corresponding fluorescence secondary antibodies (Jackson ImmunoResearch Laboratories) were applied for 1 hour. Once mounted with PBS, the slides were imaged by confocal microscopy.

### ChIP-qPCR.

ChIP-qPCR was performed using the Agarose ChIP kit (26156, Thermo Fisher Scientific) following the manufacturer’s instructions. Immunoprecipitation was performed using a valid RUNX2 chip antibody (12556S, Cell Signaling Technology). The RNA polymerase II antibody as a positive control was provided in the kit. The primers used for PCR were as follows: mouse-*Slc20a1* forward, 5′-CCAGCTCACCCGGCTAGG-3′ and reverse, 5′-GAGGTGAAAACACGCAACGC-3′; the *Gapdh* primer was provided in the kit.

### Western blot analysis.

Western blot analysis of cell lysates was performed as described previously (95, 96). The antibodies used were as follows: OPN (1:1,000, AF808, R&D Systems); SLC20a1 PiT1 (1:1,000, 12423-1-AP, Proteintech); Slc20a1PiT1 (1:200, GTX64727, GeneTex); RUNX2 (1:1,000, 12556S, Cell Signaling Technology); p-RUNX2 (1:1000, PA5-105642, Thermo Fisher Scientific); PDGFRβ (1:200, 3169S, Cell Signaling Technology); p-PDGFRβ (1:200, 3161S, Cell Signaling Technology); p-ERK (1:1,000, 9101, Cell Signaling Technology); ERK (1:1,000, 4695, Cell Signaling Technology); p-AKT (1:2,000, 4060, Cell Signaling Technology); AKT (1:1,000, 4691, Cell Signaling Technology); GAPDH (1:2,000, 5174S, Cell Signaling Technology); and β-actin (1:2,000, 93473SF, Cell Signaling Technology). All blots were visualized with Super-Signal West Pico Femto Maximum Sensitivity Substrate (34094, Thermo Fisher Scientific). The relative density was determined using ImageJ. See complete unedited blots in the supplemental material.

### qRT-PCR and osteogenesis real-time profiler PCR arrays.

PowerUp SYBR Green Master mix (A25778, Thermo Fisher Scientific) was used to amplify the cDNA product. Target gene expression was normalized by the *Gapdh* or *Actb* housekeeping gene, and fold changes were calculated with the 2^–ΔΔCt^ method. The following primers were used: *Alpl* forward, 5′-GGGACGAATCTCAGGGTACA-3′, reverse, 5′-AGTAACTGG GGTCTCTCTCTTT-3′; *Spp1* forward, 5′-TGGCAGCTCAGAGGAGAAG AAGC-3′, reverse, 5′-GGGTCAGGCACCAGCCATGTG-3′; *Slc20a1* forward, 5′-TTTGACAAACTTCCTCTG TGGG-3′, reverse, 5′-GGACTTTCAGACGGACTAGACTT-3′; *Runx2* forward, 5′-AGAGT CAGATTACAGATCCCAGG-3′, reverse, 5′-TGGCTCTTCTTACTGAGAGAGG-3′; *Bglap* forward, CAGACACCATGAGGACCATC, reverse, GGACTGAGGCTCTGTGAGGT; and *Gapdh* forward, 5′-AATGTGTCCGTCGTGGATCTGA-3′, reverse, 5′-AGTGTAGCCCAAGATGCC CTTC-3′. The Mouse Osteogenesis RT2 Profiler PCR Array kit (QIAGEN) was used for the osteogenesis real-time profiler PCR arrays. Briefly, cDNA was added to the mouse osteogenic array (PAMM-026Z, QIAGEN) according to the manufacturer’s instructions. The data analysis procedure was from the manufacturer’s handbook. All gene changes are displayed on a scatterplot, the threshold of fold change threshold is 1.5, and the *P* values for all genes listed are below 0.05.

### Basic processing and gene enrichment analysis of single-cell transcriptomic data.

A scRNA-Seq data set was downloaded from the GEO database and contained single-cell transcriptome profiling of aging mouse brain (GEO GSE129788) (41). Basic data processing and visualization of scRNA-Seq data were performed with the Seurat package (version 4.1.1) in R (version 4.0.3) (88, 97, 98). Processed data and metadata, which included author-defined cell-type annotation and *t*-distributed stochastic neighbor embedding (*t*-SNE), were downloaded from the Broad Institute’s web portal (https://singlecell.broadinstitute.org/single_cell/study/SCP263/aging-mouse-brain)for aging mouse brain, where raw data processing, quality control parameters, and downstream analysis steps are detailed in the original work (41). Differential gene expression analysis was performed in Seurat using the FindAllMarkers function with the Wilcox rank-sum method. Plots were generated with ggplot2 (version 3.3.6) and Seurat using the DotPlot and VlnPlot functions.

### Statistics.

The scRNA-Seq data were analyzed with the Wilcoxon rank-sum test. Data were analyzed using GraphPad Prism 9 (GraphPad Software). All the data are shown as the mean ± SEM. An unpaired, 2-tailed Student’s *t* test was used to evaluate the statistical difference between 2 groups. For multiple groups, 1-way ANOVA analysis was used to evaluate statistical differences. A *P* value less than 0.05 was considered significant.

### Study approval.

All animal studies described in this work were conducted under protocol MO21M37, approved by the IACUC of The Johns Hopkins University.

### Data availability.

scRNA-Seq data were deposited in the GEO database (GEO GSE129788), and microarray data were deposited in the GEO database (GEO GSE243144). The data that support the findings of this study are available within the article and in the supplemental materials or from the corresponding author upon reasonable request. Values for all data points in graphs are reported in the Supplemental Supporting Data Values file.

## Author contributions

JW and MW designed the experiments, analyzed results, and wrote the manuscript. JW carried out most of the experiments. KN helped with scRNA-Seq data analysis. ZW helped with SWI and phase-mapping scanning and analysis. CLF, GL, KS, and KPS helped with some experiments. XC proofread the manuscript. MW supervised the experiments.

## Supplementary Material

Supplemental data

Supporting data values

## Version 1 10/10/2023

In-Press Preview

## Version 2 12/01/2023

Electronic publication
