# Supplementary material for: Bone-derived PDGF-BB drives brain vascular calcification in male mice
Source: J Clin Invest. 2023 Dec 1;133(23):e168447. doi: 10.1172/JCI168447 (PMC10688993; doi:10.1172/JCI168447)

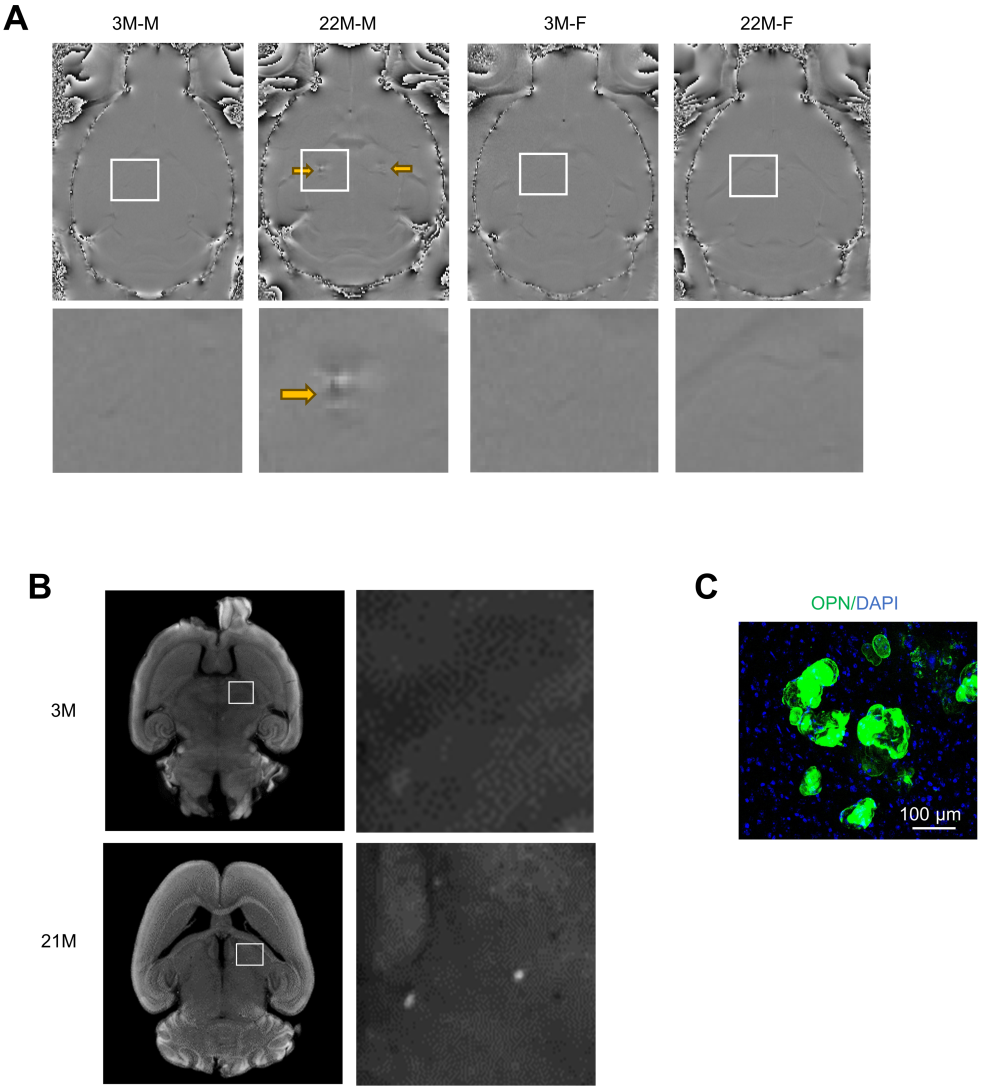

Supplemental Figure 1. Characterization of brain calcification. (**A**) SWI phase maps of the mouse brain were obtained, with yellow arrows indicating areas of calcification. Boxed areas are shown at a higher magnification in corresponding panels at the bottom. n=5. (**B**) Representative micro-CT images of the whole brain from 3- and 22-month-old mice. n=3. (**C**) A representative image of immunofluorescence staining of OPN in the thalamus region in aged mice after the SWI scanning to confirm the presence of calcified nodule.

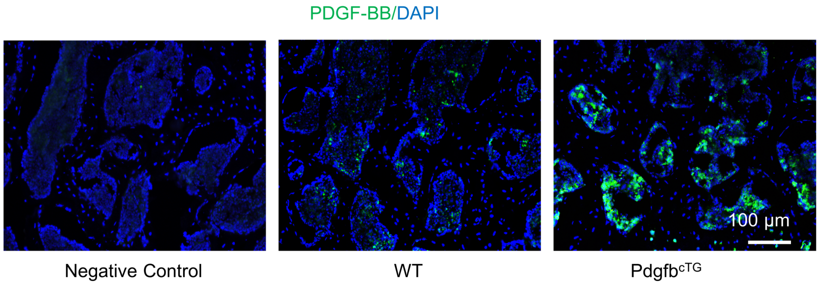

Supplemental Figure 2. Increased PDGF-BB in bone/bone marrow of Pdgfb^{cTG} mice. Immunofluorescence staining of PDGF-BB was performed using frozen femur tissue sections obtained from 3-month-old Pdgfb^{cTG} mice and wild type littermates (WT). n=3. No primary antibody was added in the Negative Control group.

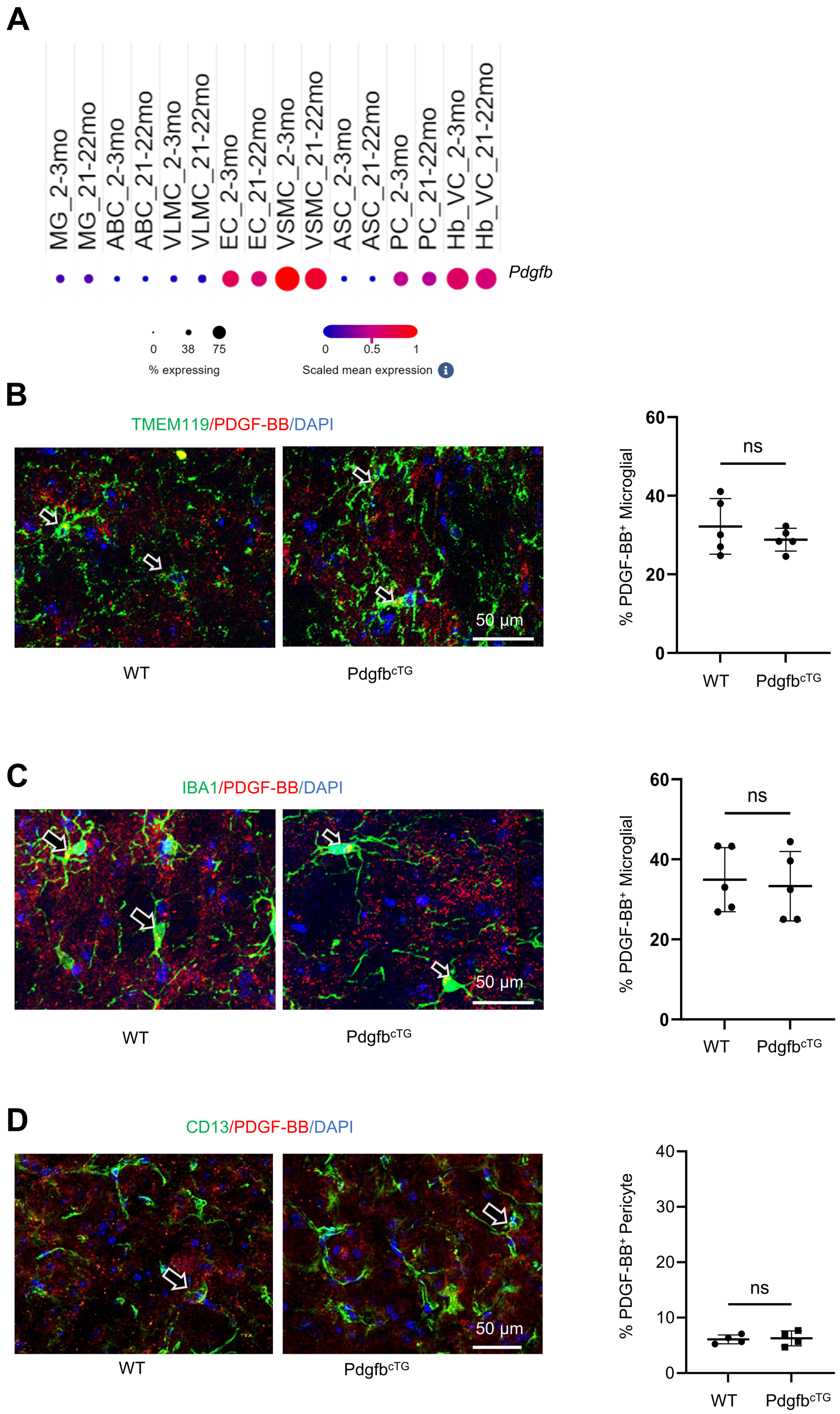
A
MG_2-3mo
MG_21-22mo
ABC_2-3mo
ABC_21-22mo
VLMC_2-3mo
VLMC_21-22mo
EC_2-3mo
EC_21-22mo
VSMC_2-3mo
VSMC_21-22mo
ASC_2-3mo
ASC_21-22mo
PC_2-3mo
PC_21-22mo
Hb_VC_2-3mo
Hb_VC_21-22mo
Pdgfb
0 38 75
% expressing
0 0.5 1
Scaled mean expression
B
TMEM119/PDGF-BB/DAPI
50 μm
WT
PdgfbcTG
% PDGF-BB+ Microglial
ns
C
IBA1/PDGF-BB/DAPI
50 μm
WT
PdgfbcTG
% PDGF-BB+ Microglial
ns
D
CD13/PDGF-BB/DAPI
50 μm
WT
PdgfbcTG
% PDGF-BB+ Pericyte
ns

Supplemental Figure 3. Characterization of PDGF-BB-expressing cells in brain. (**A**) Dot plot represents the expression of *Pdgfb* in young and aged mice by analysis of a single-cell RNA sequencing dataset (GSE129788) (see detailed information in Methods section). (**B**) Double immunofluorescence staining of TMEM119 and PDGF-BB was performed using frozen brain tissue sections obtained from 6-month-old $Pdgfb^{cTG}$ mice and wild type littermates (WT). Arrows: $PDGF\text{-}BB^{+}TMEM119^{+}$ double positive cells. n=5. Percentage of $PDGF\text{-}BB^{+}$ microglia cells in thalamus region was shown in the right panel. (**C**) Double immunofluorescence staining of IBA1 and PDGF-BB was performed using frozen brain tissue sections obtained from 6-month-old $Pdgfb^{cTG}$ mice and wild type littermates (WT). Arrows: $PDGF\text{-}BB^{+}IBA1^{+}$ double positive cells. n=5. Percentage of $PDGF\text{-}BB^{+}$ microglia cells in thalamus region was shown in the right panel. (**D**) Double immunofluorescence staining of CD13 and PDGF-BB was performed using frozen brain tissue sections obtained from 3-month-old $Pdgfb^{cTG}$ mice and wild type littermates (WT). Arrows: $PDGF\text{-}BB^{+}CD13^{+}$ double positive cells. n=4. Percentage of $PDGF\text{-}BB^{+}$ pericyte cells in thalamus region was shown in the right panel. Unpaired two-tailed Student's t test was used to perform two group comparison (**B-D**).

A

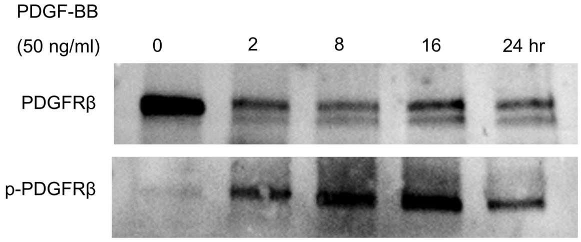

B

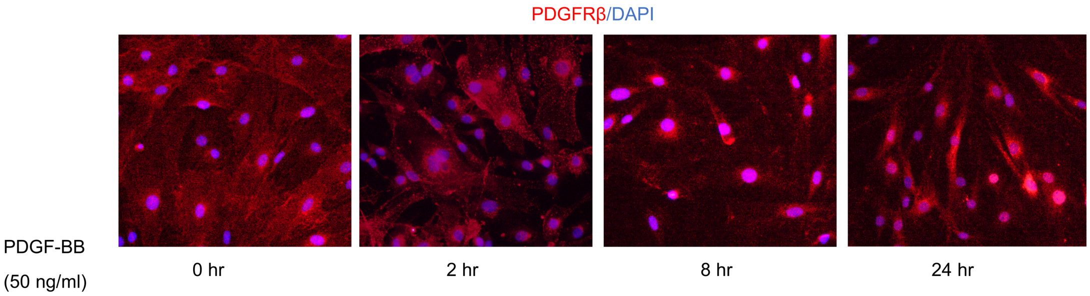

Supplemental Figure 4. PDGF-BB treatment reduces cell surface PDGFRβ expression in pericytes. Brain pericytes were subjected to treatment with 30 ng/ml PDGF-BB for different time periods. (**A**) Western blot analysis was conducted to evaluate the protein expression levels of phosphorylated PDGFRβ (p-PDGFRβ) and total PDGFRβ (t-PDGFRβ) in primary pericytes. (**B**) Immunocytochemistry staining was performed using antibodies specific for PDGFRβ.

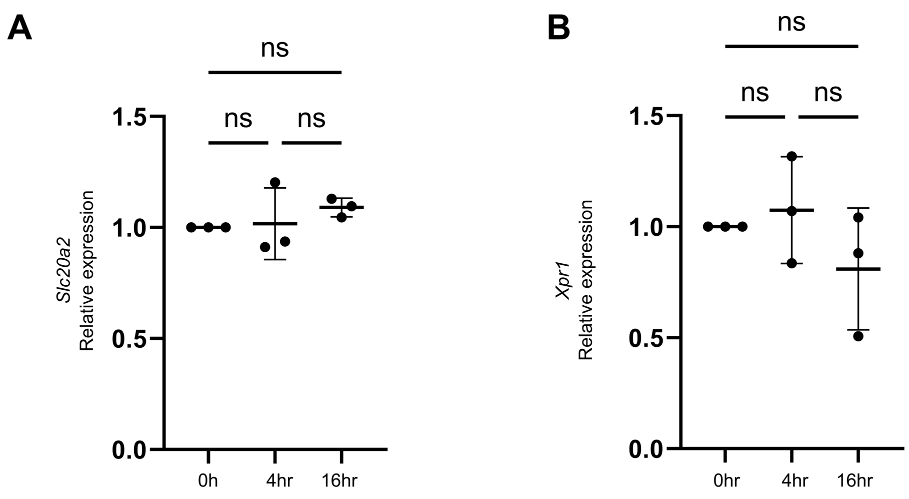

Supplemental Figure 5. ***Slc20a2*** **and** ***Xpr1*** **did not overexpress after PDGF-BB treatment.** (**A and B**) The quantitative real-time PCR analysis of *Slc20a2* (**A**) and *Xpr1* (**B**) mRNA expression in isolated mouse brain microvessel treated with 50 ng/ml PDGF-BB at different time periods**.** Ordinary one-way ANOVA was used to perform multiple group comparisons (**A and B**).

OPN Full unedited blot for Figure 2G

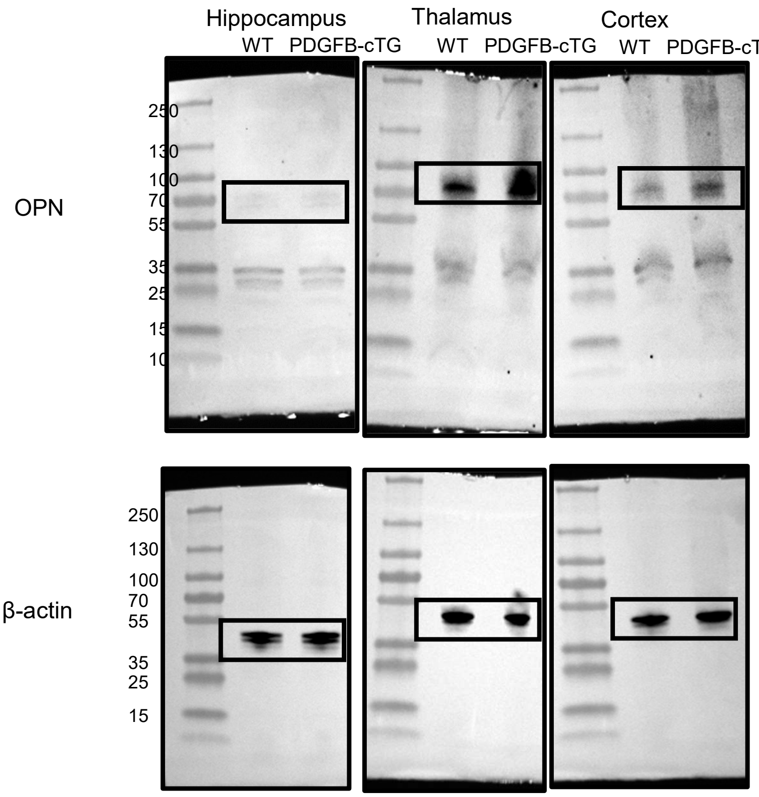

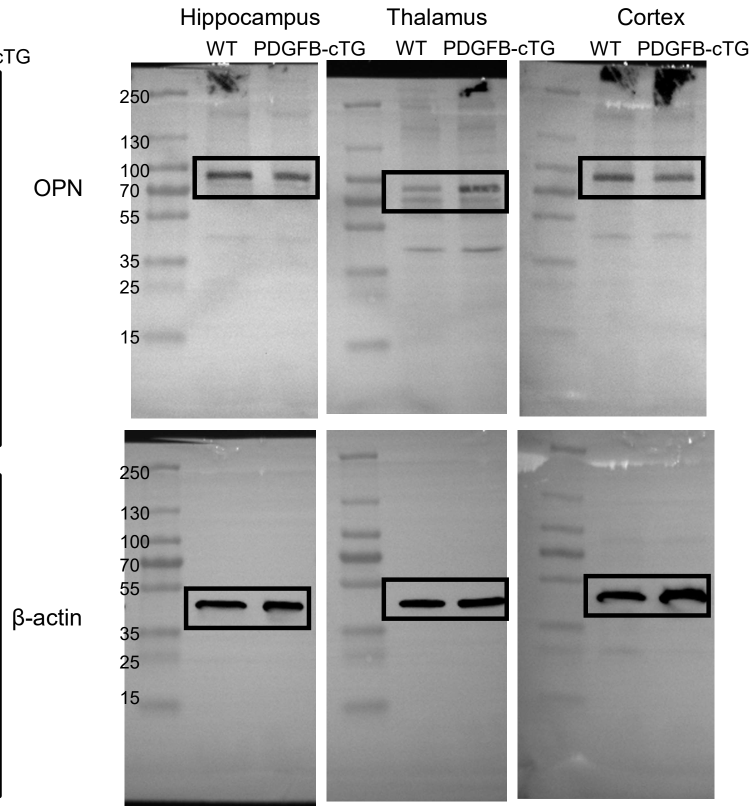

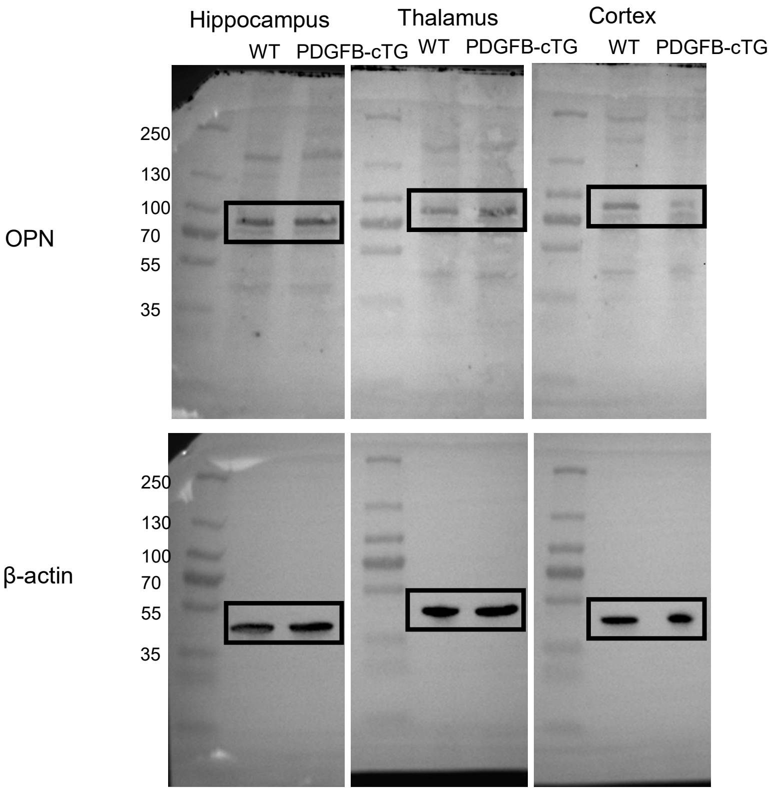

OPN Full unedited blot for Figure 2G

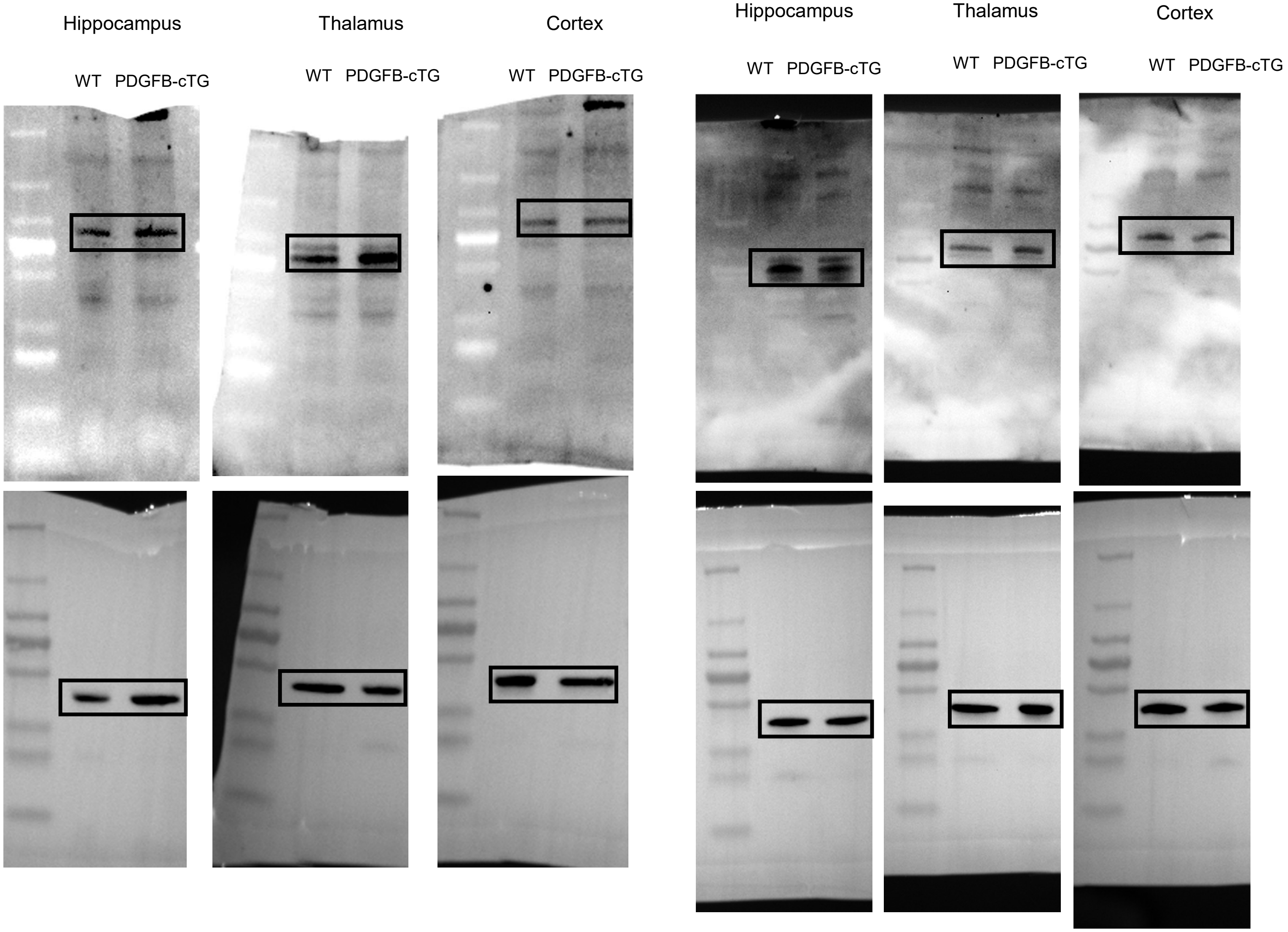

p-PDGFRβ, t-PDGFRβ, p-ERK and t-ERK full unedited blot for Figure 6A

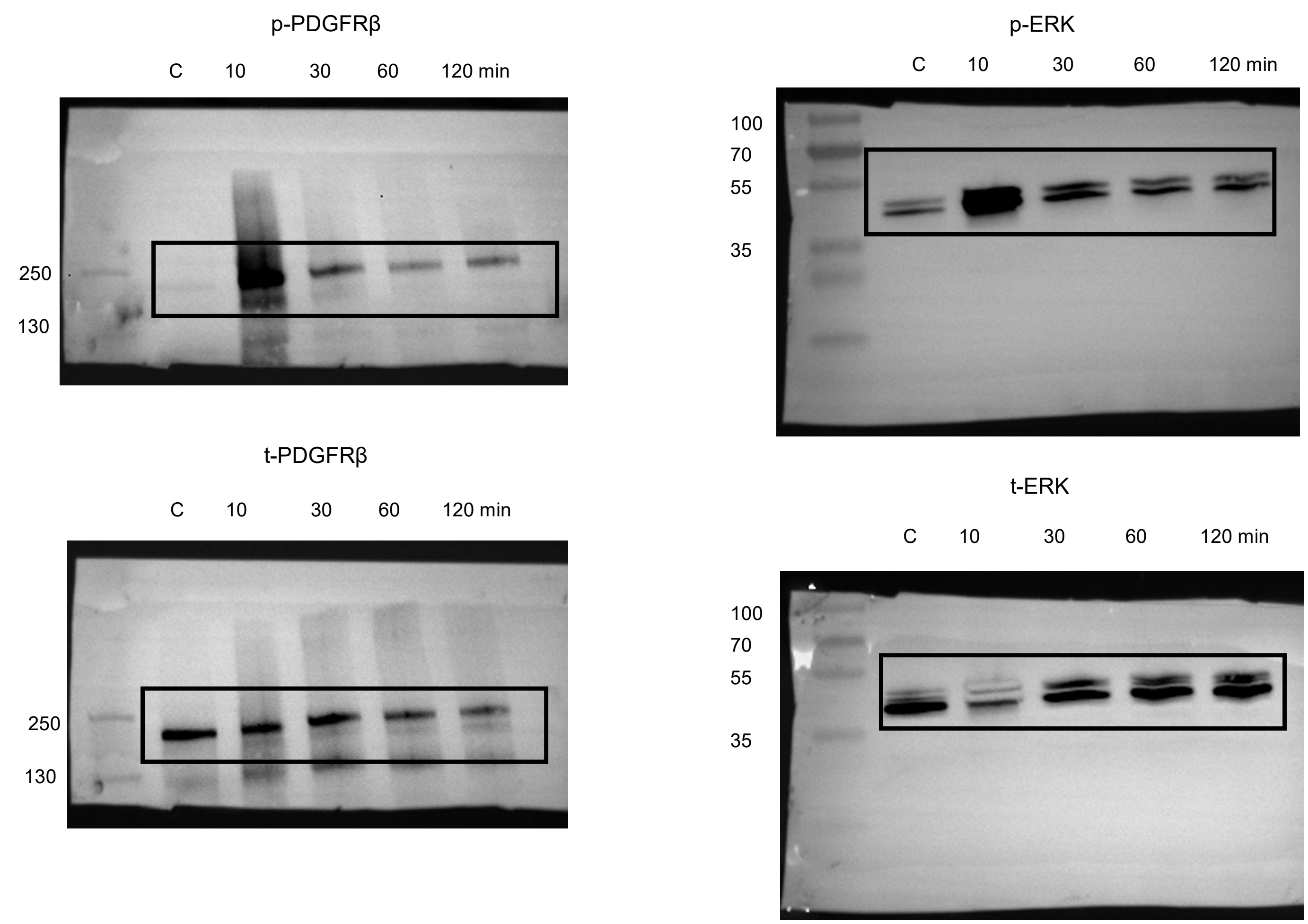

p-PDGFRβ, t-PDGFRβ, p-ERK and t-ERK full unedited blot for Figure 6C

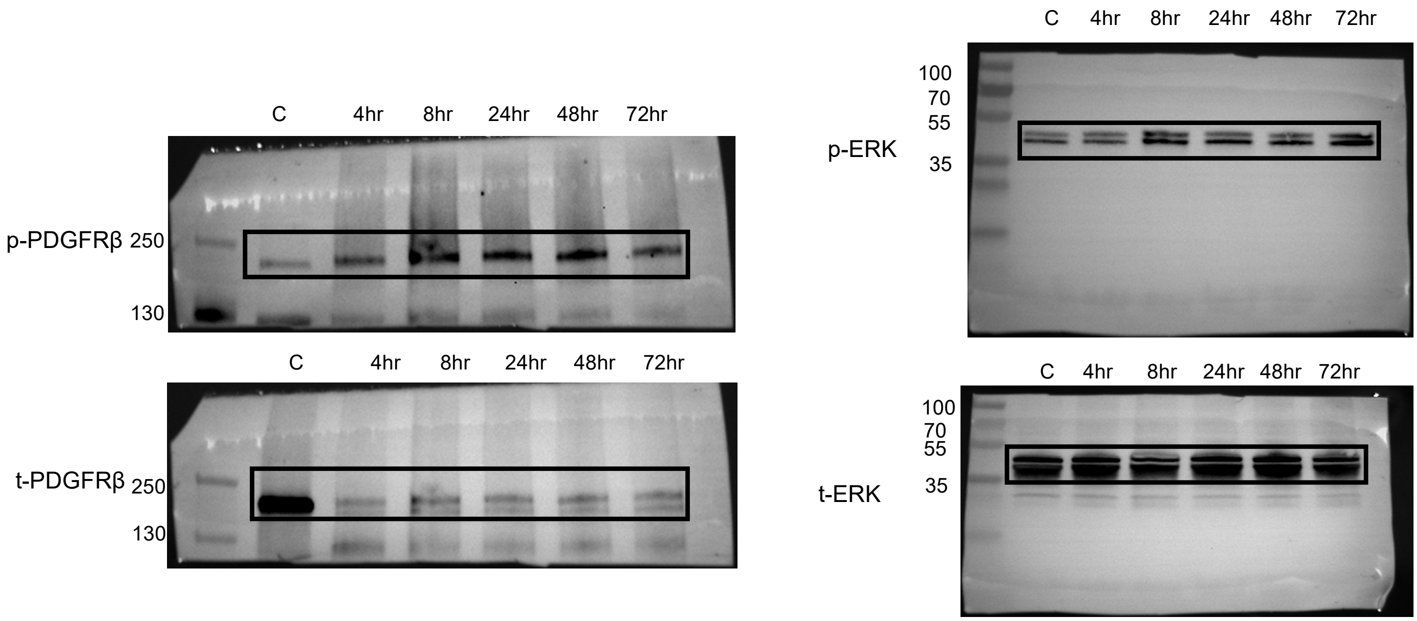

OPN RUNX2 Full unedited blot for Figure 6E

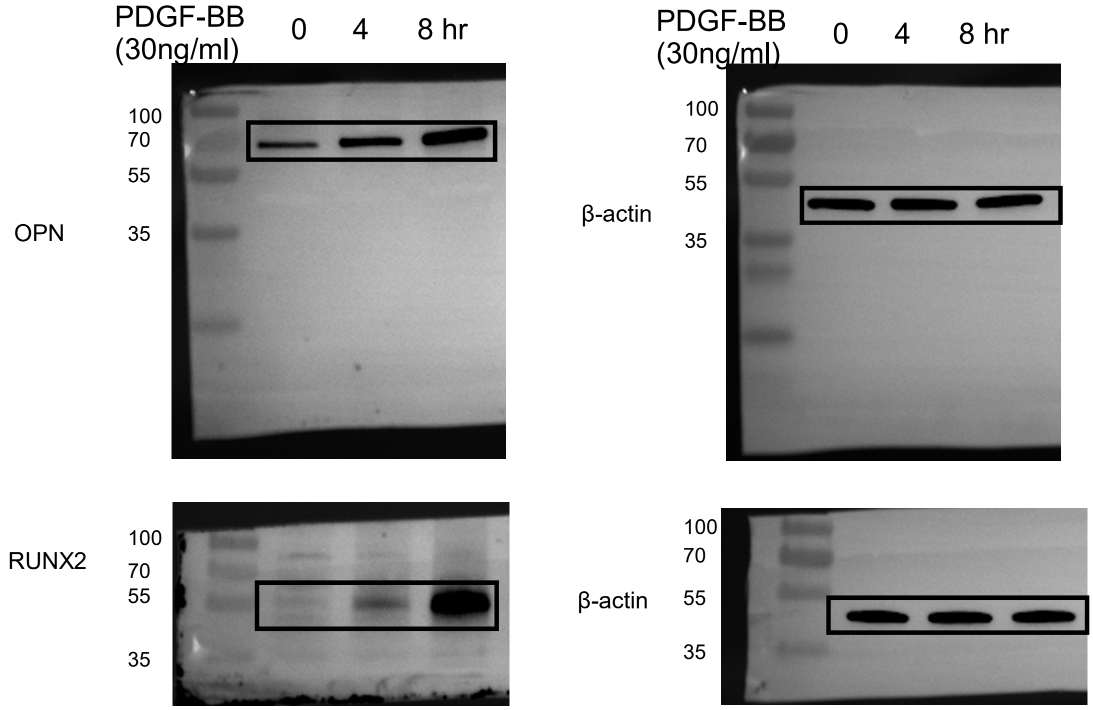

PiT1 Full unedited blot for Figure 7B

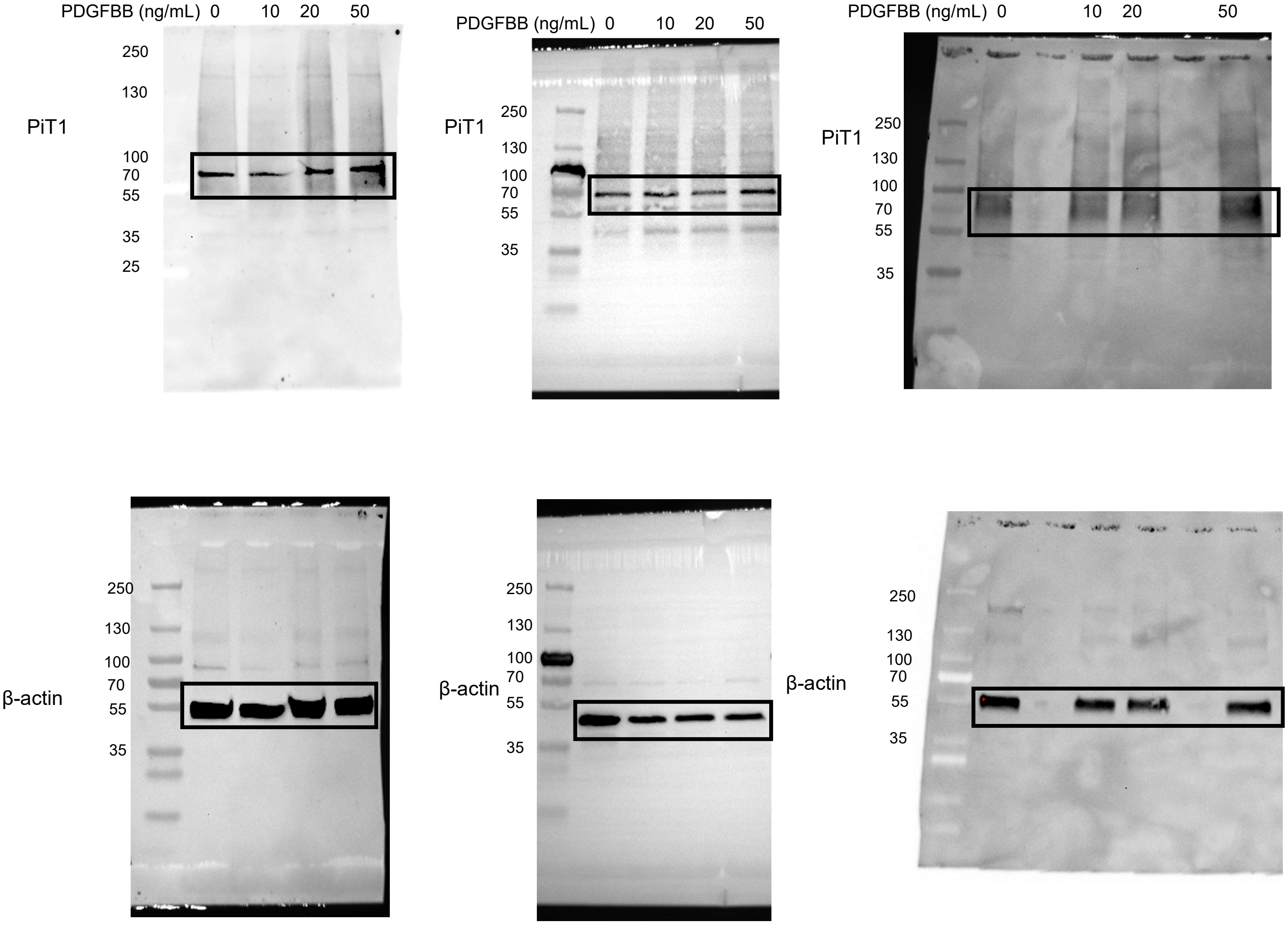

Astrocyte PiT1 Full unedited blot for Figure 7H

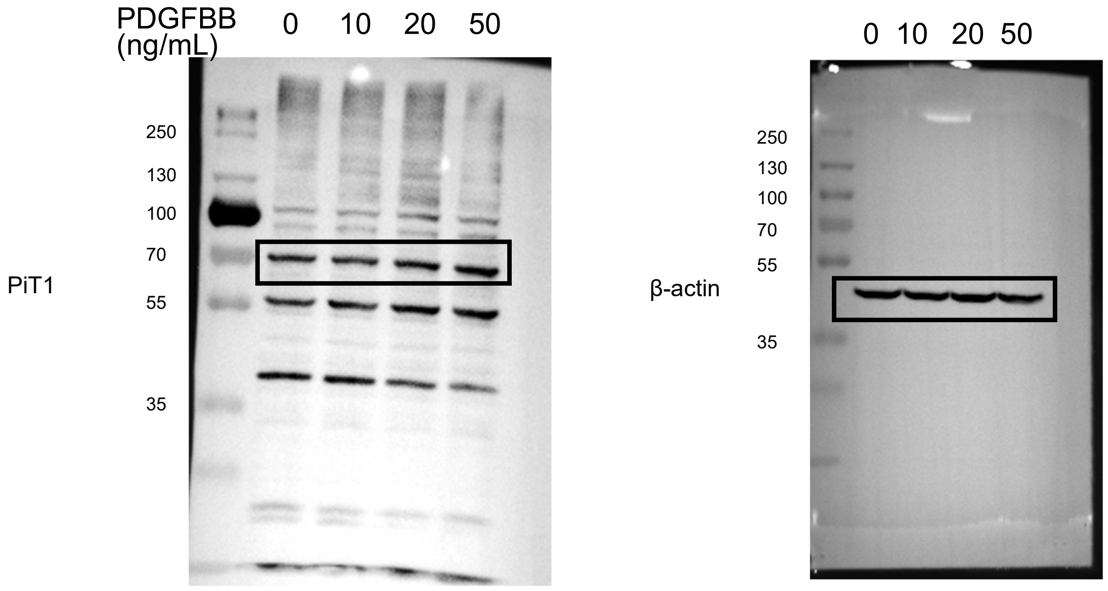

Pericyte PiT1 Full unedited blot for Figure 7I

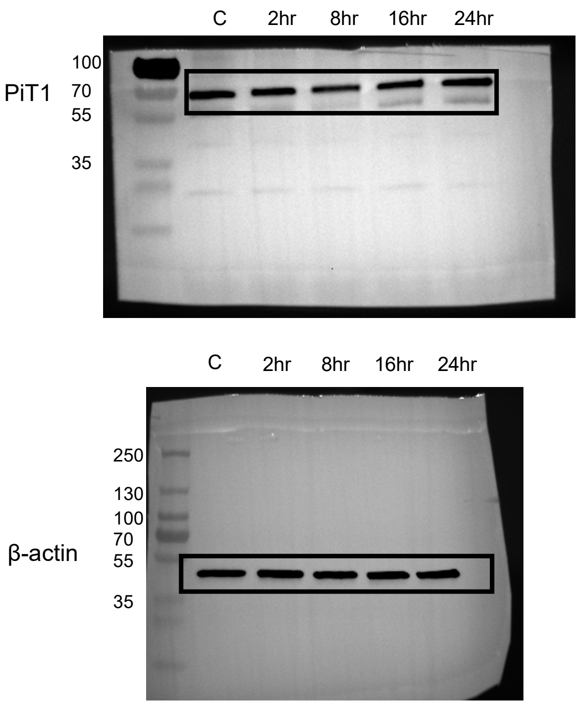

p-runx 2 Full unedited blot for Figure 8C

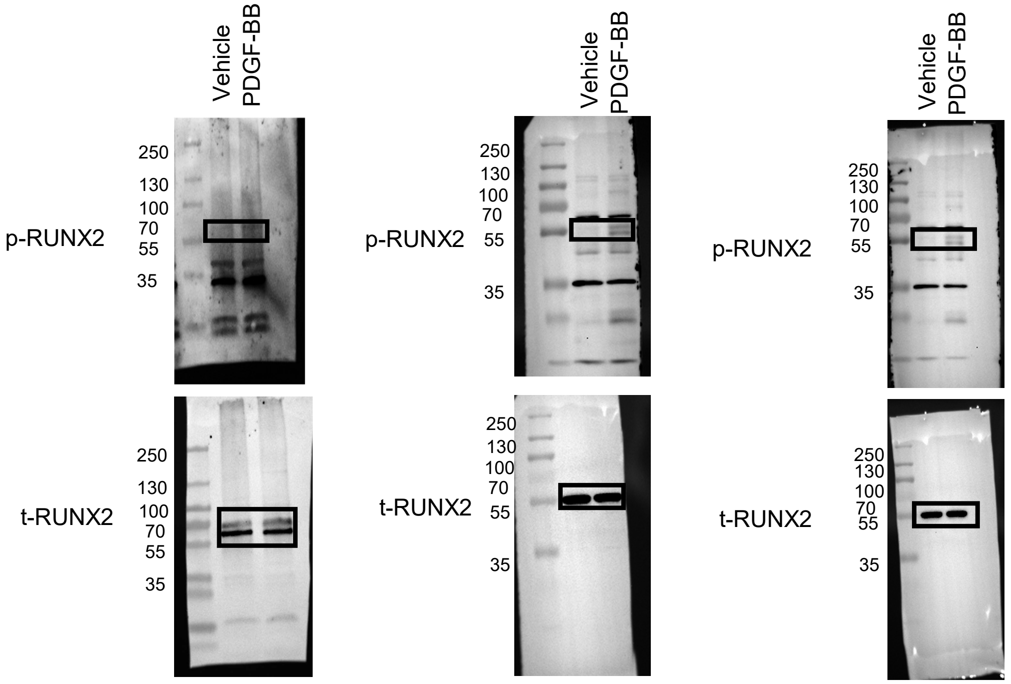

t-ERK, p-ERK, p-AKT, AKT Full unedited blot for Figure 8F

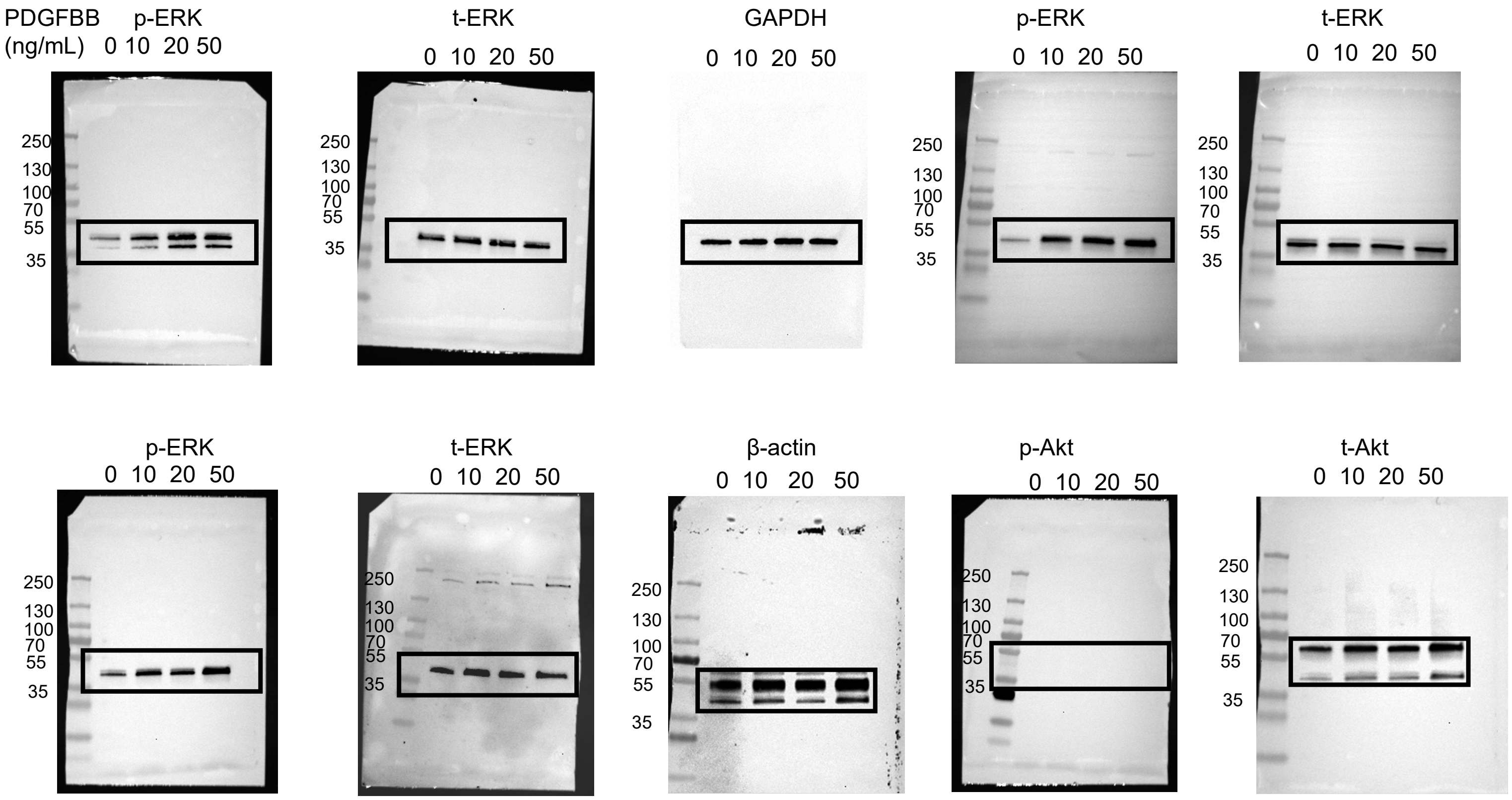

Chip assay Full unedited gel for Figure 8B

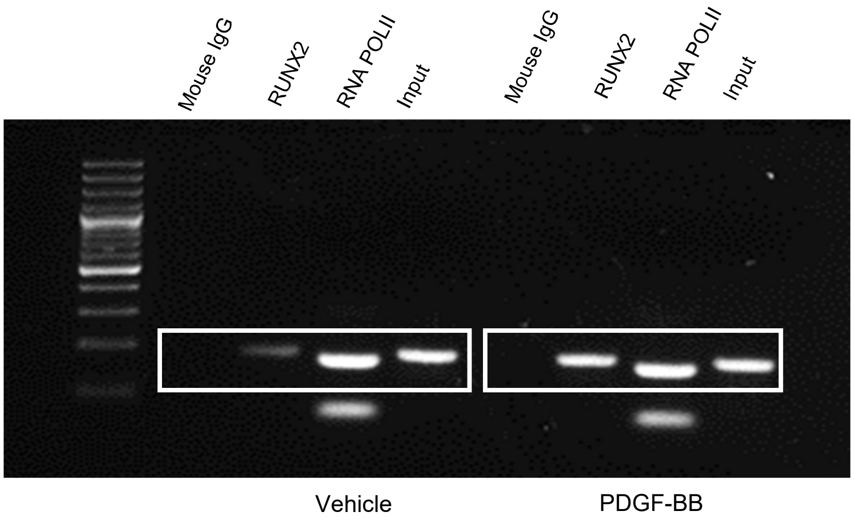

p-PDGFRβ, t-PDGFRβ full unedited blot for supplement Figure 4A

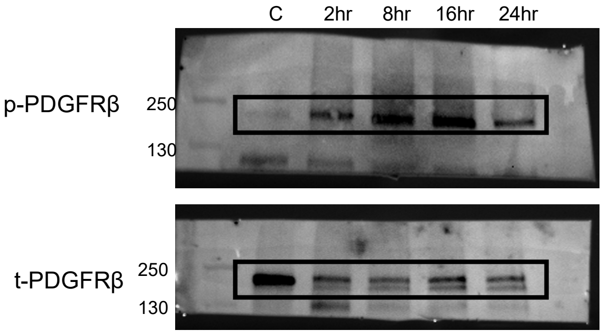

Supplement: Supplemental data [file jci-133-168447-s178.pdf]
